# Time-varying SMART design and data analysis methods for evaluating adaptive intervention effects

**DOI:** 10.1186/s12874-016-0202-7

**Published:** 2016-08-30

**Authors:** Tianjiao Dai, Sanjay Shete

**Affiliations:** 1 Department of Biostatistics, The University of Texas MD Anderson Cancer Center, 1400 Pressler Dr, FCT4.6002, Houston, TX 77030 USA; 2 Department of Epidemiology, The University of Texas MD Anderson Cancer Center, Houston, TX 77030 USA

**Keywords:** Adaptive interventions, Sequential multiple assignment randomized trial (SMART), Time-varying mixed effects model (TVMEM), Longitudinal model, Joint model

## Abstract

**Background:**

In a standard two-stage SMART design, the intermediate response to the first-stage intervention is measured at a fixed time point for all participants. Subsequently, responders and non-responders are re-randomized and the final outcome of interest is measured at the end of the study. To reduce the side effects and costs associated with first-stage interventions in a SMART design, we proposed a novel time-varying SMART design in which individuals are re-randomized to the second-stage interventions as soon as a pre-fixed intermediate response is observed. With this strategy, the duration of the first-stage intervention will vary.

**Methods:**

We developed a time-varying mixed effects model and a joint model that allows for modeling the outcomes of interest (intermediate and final) and the random durations of the first-stage interventions simultaneously. The joint model borrows strength from the survival sub-model in which the duration of the first-stage intervention (i.e., time to response to the first-stage intervention) is modeled. We performed a simulation study to evaluate the statistical properties of these models.

**Results:**

Our simulation results showed that the two modeling approaches were both able to provide good estimations of the means of the final outcomes of all the embedded interventions in a SMART. However, the joint modeling approach was more accurate for estimating the coefficients of first-stage interventions and time of the intervention.

**Conclusion:**

We conclude that the joint modeling approach provides more accurate parameter estimates and a higher estimated coverage probability than the single time-varying mixed effects model, and we recommend the joint model for analyzing data generated from time-varying SMART designs. In addition, we showed that the proposed time-varying SMART design is cost-efficient and equally effective in selecting the optimal embedded adaptive intervention as the standard SMART design.

**Electronic supplementary material:**

The online version of this article (doi:10.1186/s12874-016-0202-7) contains supplementary material, which is available to authorized users.

## Background

Sequential, multiple assignment, randomized trial (SMART) designs and their analysis are being used to construct high-quality adaptive interventions that can be individualized by repeatedly adjusting the intervention(s) over time on the basis of individual progress [1–5]. The SMART design was pioneered by Murphy, building on the work of Lavori and Dawson [6, 7]. SMART designs involve an initial randomization of individuals to different intervention options, followed by re-randomization of some or all of the individuals to another set of available interventions at the second stage. At subsequent stages, the probability and type of intervention to which individuals are re-randomized may depend on the information collected from the previous stage(s) (e.g., how well the patient responded to the previous treatment; adherence to treatment protocol). Thus, there can be several adaptive interventions embedded within each SMART design. This allows for testing the tailoring variables and the efficacy of the interventions in the same trial. There are several practical examples of SMART studies that have been conducted (e.g., the CATIE trial [8] for antipsychotic medications in patients with schizophrenia, STAR*D for the treatment of depression, [9, 10] and phase II trials at MD Anderson for treating cancer [11]). The goal of these studies is to optimize the long-term outcomes by incorporating the participant’s characteristics and intermediate outcomes evaluated during the intervention [12, 13].

An example of a two-stage SMART design is a study that characterized cognition in nonverbal children with autism [14]. To improve verbal capacity, participants were initially randomized to receive either a combination of behavioral interventions (Joint Attention Symbolic Play Engagement and Regulation (JASPER) + Enhanced Milieu Training (EMT)) or an augmented intervention (JASPER + EMT+ speech-generating device [SGD]). Children were assessed for early response versus slow response to the first-stage treatment at the end of 12 weeks. The second-stage interventions, administered for an additional 12 weeks, were chosen on the basis of the response status (only slow responders to JASPER + EMT were re-randomized to intensified JASPER + EMT or received the augmented JASPER + EMT + SGD; slow responders to JASPER + EMT + SGD received intensified treatment; all early responders continued on the same intervention). There were three pre-fixed assessment time points: at 12 weeks, 24 weeks and 36 weeks (follow-up), which were the same for all participants in the study. Compared to multiple, one-stage-at-a-time, randomized trials, SMART designs provide better ability to compare the impact of a sequence of treatments, rather than examining each piece individually. For example, a SMART allows us to detect possible delayed effects in which an intervention at a previous stage has an effect that is less likely to occur unless it is followed by a particular subsequent intervention option. The typical modeling approach for the SMART design as described by Nahum-Shani et al. includes the indicators of intervention at each stage as covariates and thus accounts for the delayed effects on the final response. In order to develop a sequence of best decision rules for each individual, various statistical learning methods of estimating the optimal dynamic treatment regimens have been proposed, among which Q-learning has been developed for assessing the relative quality of the intervention options and estimating the optimal (i.e., most effective) sequence of decision rules with linear regression. For a two-stage SMART, the Q-learning approach controls for the optimal second-stage intervention option when assessing the effect of the first-stage intervention, and reduces the potential bias resulting from unmeasured causes of both the tailored variables and the primary outcome. A similar approach for deriving the optimal decision rules for SMART is A-learning, which is more robust to model misspecification than Q-learning for consistent estimation of the optimal treatment regime [15]. Zhao et al. introduced the two learning methods of BOWL and SOWL, [16] which are based on directly maximizing over all dynamic treatment regimens (DTRs) a nonparametric estimator of the expected long-term outcome. As an alternative to the above learning approaches, Zhang et al. [17] proposed a robust estimation of the optimal dynamic treatment regimens for sequential treatment decisions, which maximizes a doubly robust augmented inverse probability weighted estimator for the population mean outcome over a restricted class of regimes. All these approaches model the outcomes of interest as dependent variables, and for the predictor variables, they model the main and interacting effects of the intervention options at each stage and the baseline individual characteristics. The amount of time an intervention is administered, however, is not explicitly modeled, although it can be used as a covariate in these regressions.

There are examples of SMART designs in which a participant is assessed at several pre-fixed time points during the first-stage treatment and once he/she meets an assigned criterion for response status, he/she is re-randomized to the second stage of treatment. Such a SMART design has been applied to develop a dynamic treatment regime for individuals with alcohol dependence using the medication naltrexone [2, 18, 19]. At the beginning of the study, patients were randomized to either a stringent or a lenient criterion for early non-response. Initially, all patients received naltrexone. Starting at the end of the second week, patients who showed early response were assessed weekly for eight weeks, and those who met the assigned criterion for non-response were assigned to the second stage randomization in that week; whereas the responders were re-randomized at week eight. Another example of using a SMART design to evaluate multiple, fixed time points is the study of pharmacological and behavioral treatments for children with ADHD, where children were assessed monthly for response or non-response [19–23]. In addition, Lu et al. [19] developed repeated-measures piecewise marginal models for comparing embedded treatments in such SMART designs with multiple evaluations at fixed time points. In these studies, subjects were assessed at fixed time points; thus, the time of treatment takes values along a finite set of time points.

Although, SMART designs with outcome assessments at fixed time points exist, there are advantages to administering a drug as soon as an individual achieves an intermediate response. For example, the smoking cessation drugs varenicline and bupropion can increase the risk of psychological side effects such as unusual changes in behavior, hostility, agitation, depressed mood and suicidal thoughts [24–26]. In addition, varenicline costs approximately $300 per month. Therefore, allowing the duration of treatment to vary among participants for one or more stages of the study may reduce the side effects and costs associated with the interventions. For such time-varying SMART designs, the duration of treatment plays an important role in decision making, and including it in the analysis may increase the power of the study and better serve our goal of analysis. To further extend the assignment strategies discussed in the above examples and utilize the information contained in the treatment duration, in this paper, we proposed a novel time-varying SMART design, which enables us to more efficiently assign different intervention options as soon as an individual achieves a set of intermediate response goals. Therefore, the time of treatment is a continuous random variable for each individual that can take any value on a subset of the positive real line, and is treated as an endogenous variable. The existing statistical methods are inappropriate for analyzing data obtained from such a time-varying SMART design. Therefore, to fully utilize the potential of this type of time-varying SMART design in making more efficient decisions, we also proposed two analytic approaches that can be used to analyze data from such a time-varying SMART design. The first approach is a linear mixed model with time-varying fixed effects [27, 28], which is in fact a piecewise linear model. The second approach incorporates a joint modeling method in which a survival model is fitted jointly with the linear mixed model [29]. We performed simulations to evaluate the statistical properties of both methods. Our simulation results showed that both methods estimated the expected final outcome for each embedded adaptive intervention in such design accurately, but the joint-modeling method provided better estimates for certain parameters in the model.

To compare the power and cost efficiency of the time-varying SMART design to those of an analogous standard SMART design, we simulated two trials with identical sample sizes and intervention effects using (a) the time-varying SMART design and (b) the standard SMART design. These simulations showed that the time-varying SMART design is cost-efficient and has power similar to that of the standard SMART design in selecting the optimal embedded adaptive intervention.

## Methods

### Proposed time-varying SMART design

**Fig. 1:**
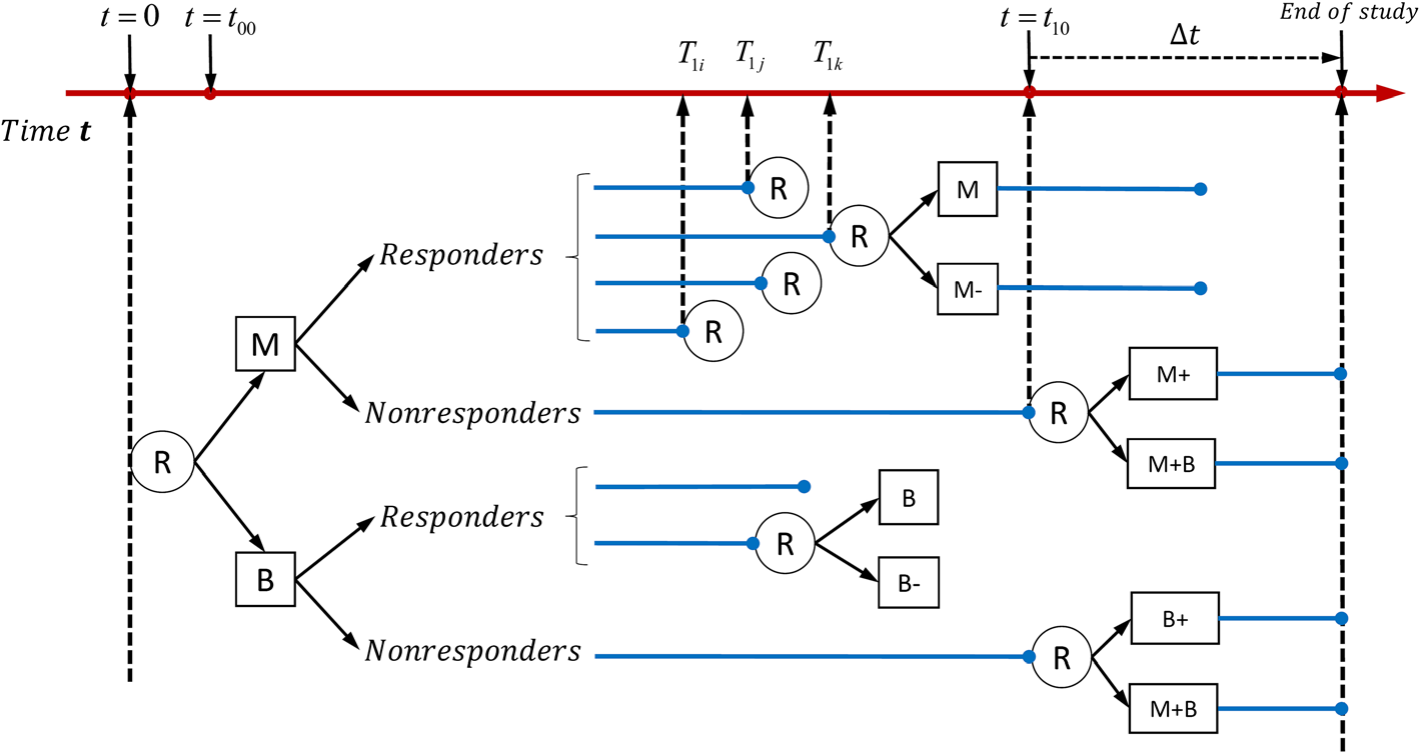
Example of time-varying two-stage SMART design with equal probability allocation: each participant is randomized twice

**Fig. 2:**
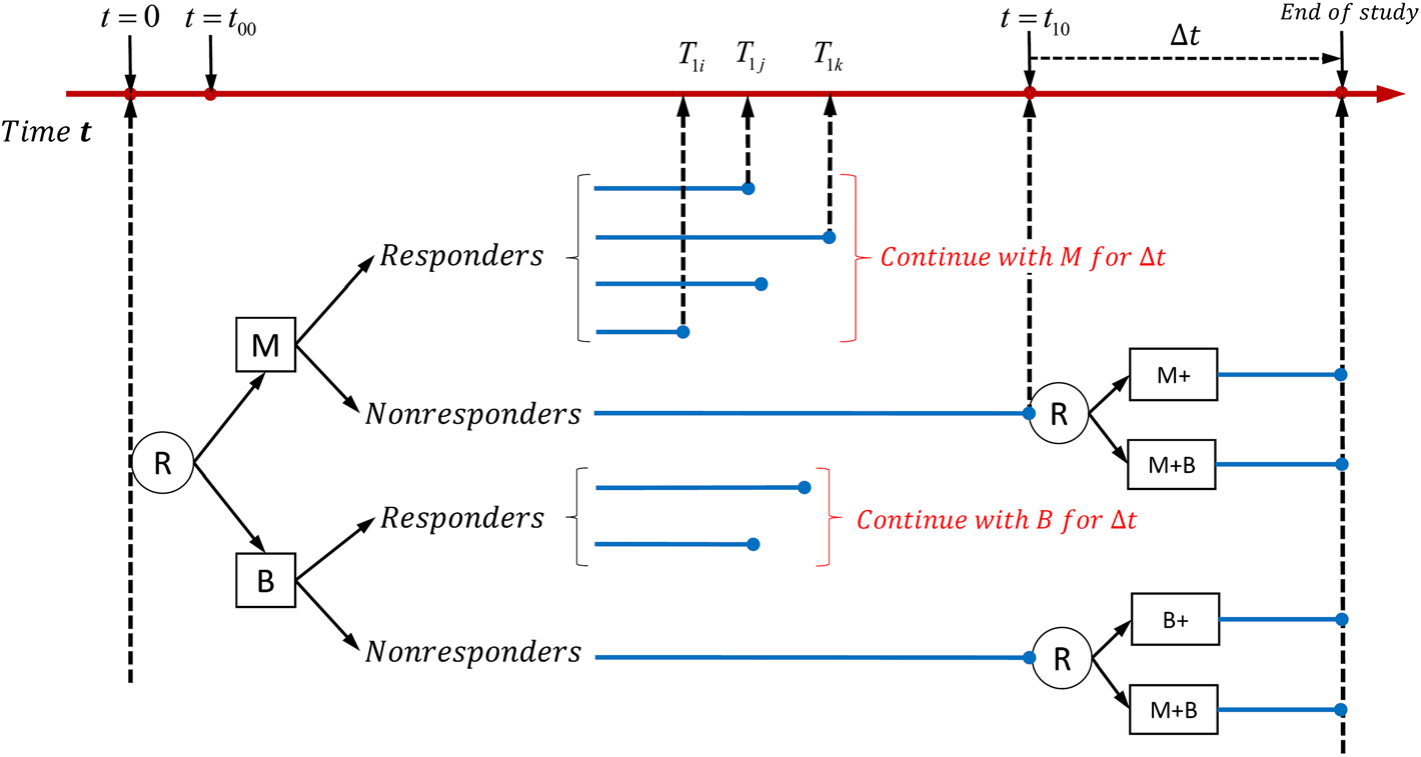
Example of time-varying two-stage SMART design with unequal probability allocation: only non-responders are re-randomized in the second stage

Figures 1 and 2 illustrate the proposed time-varying designs. Both two-stage time-varying SMARTs were designed to provide data regarding how the intensity and combination of two types of interventions might be adapted to a subject’s progress in a cost- and time-efficient manner.

In the first example (see Fig. 1), suppose medication (M) and behavioral intervention (B) are two initial intervention options for individuals who are heavy smokers (e.g., those who smoke more than or equal to 25 cigarettes per day). The number of cigarettes a subject smokes per day is the outcome of interest and is measured at the beginning of the study, at several intermediate time points and at the end of the study. Let *Y*_0_ denote the number of cigarettes a subject smoked per day at the beginning of the study (t = 0). Subjects are randomly assigned to the medication or the behavioral interventions at the beginning of the study. Monitoring the outcome of interest begins at a pre-fixed time point (e.g. one week after the initial randomization and is denoted as *t*_00_) after the initial intervention is implemented, and *t*_10_ denotes the time point at which those who did not respond to a first-stage intervention are re-randomized. A subject is considered a responder to the first-stage intervention if there is a significant decrease in the number of cigarettes he/she smoked per day (e.g., the decrease in the number of cigarettes smoked per day is above a pre-fixed threshold, C) at an intermediate time point *T*_1_, before *t*_10_. Thus, *T*_1_ is a random variable of time and varies among responders. A subject is classified as a non-responder if the decrease in the number of cigarettes he or she smoked per day by *t*_10_ is below C. Therefore, all the non-responders are given the first-stage intervention for a fixed time period of *t*_10_ (e.g., the first month of initial interventions), which can be seen as the right-censored time point. Let *Y*_1_ denote the number of cigarettes smoked per day at the end of the first-stage intervention.

An indicator variable *δ* is defined as *δ* = *I*(*T*_1_ < *t*_10_), where *I*(⋅) is the indicator function that takes the value 1 if *T*_1_ < *t*_10_ (i.e., if the subject is a responder) and the value 0 if T_1_ ≥ t_10_ (i.e., if the subject is a non-responder). A responder is re-randomized either to continue with the first-stage intervention (M or B) or to receive the first-stage intervention at a reduced intensity (M- or B-); whereas a non-responder is re-randomized to receive the first-stage option at an increased intensity (M+ or B+) or augmented with the other type of intervention (i.e., adding a behavioral intervention for those who started with medication or adding medication for those who started with a behavioral intervention). We let all the subjects in this design stay on their second-stage interventions for a fixed time period, *Δt* (e.g., one month). Therefore, for a subject whose first-stage intervention time is *T*_1_, the total study time is *T*_1_ + *Δt*, which we denote as *T*_2_. For each participant, *Y*_2_ is the final measurement of the number of cigarettes smoked per day at *T*_2_; see Fig. 1).

The design illustrated in Fig. 2 is similar to that in Fig. 1 except that all the responders continue with their first-stage intervention options (i.e., each responder receives the same intervention after the response time point *T*_1_) in the second stage (see Fig. 2).

The adaptive interventions that are embedded within the two SMART designs in Figs. 1 and 2 are listed in Additional file 1: Tables S1 and S2.

### Analytic approach

Let *A*_1_ and *A*_2_ be the indicators of the first- and second-stage intervention options, respectively. For each individual, we observe the data (*Y*_0_, *A*_1_, *T*_1_, *Y*_1_, *A*_2_, *Y*_2_, *T*_2_, *δ*). The outcomes of interest are the longitudinal measurements *Y*_0_, *Y*_1_, and *Y*_2_, which are fitted with a linear mixed model, assuming they share the same random intercepts at the subject level. Because the intervention options and their durations change over time in this design, we first proposed a straightforward time-varying mixed effects model (TVMEM) to analyze the outcomes. In this approach, the duration of time a treatment is administered is used as a covariate in the model. Such an approach is better than the approaches that ignore the time component of the intervention (i.e., the duration of the intervention influences its effect). However, the time duration is a random variable and one may gain statistical efficiency by treating it as a dependent variable in the modeling. Therefore, we also proposed a joint-modeling approach that simultaneously postulates a linear mixed effects model for the longitudinal measurements *Y* = (*Y*_0_, *Y*_1_, *Y*_2_) and a Cox model for the survival time *T*_1_. In particular, we fit a survival submodel for *T*_1_ jointly with the previously mentioned TVMEM that will efficiently extract the information contained in *T*_1_.

### Analytic models

#### Time-varying mixed effects model of *Y* = (*Y*_0_, *Y*_1_, *Y*_2_)

A linear TVMEM is fitted to the longitudinal outcomes, with interventions and their interactions and durations included as predictors. For each individual *i* in the study, we have

1 $$ \begin{array}{c}{Y}_i(t)={m}_i(t)+{\varepsilon}_i(t)={Z}_i\eta (t)+{X}_i(t)\beta (t)+{b}_i+{\varepsilon}_i(t)\\ {}\kern3.5em ={Z}_i\eta (t)+{\beta}_0(t)+{\beta}_1(t){A}_{1i}(t)+{\beta}_2(t){A}_{2i}(t)+{\beta}_3(t)t+{\beta}_4(t){A}_{1i}(t)\cdot {A}_{2i}(t)+{b}_i+{\varepsilon}_i(t)\end{array} $$

where *m*_*i*_(*t*) is the unobserved true value of the longitudinal outcome at time point t, and *b*_*i*_ is the subject-level random effects and is assumed to be normally distributed with a mean of zero and variance of *σ*_*b*_^2^; *Z*_*i*_ is a vector of the baseline covariates (e.g., age, sex, comorbidities, etc.) with a corresponding vector of the regression coefficients *η*(*t*); *X*_*i*_(*t*) is the vector of the first-stage and second-stage intervention options, their interactions, and duration of intervention with a corresponding vector of the regression coefficients *β*(*t*). Finally, *ε*_*i*_(*t*) is the error term at time t and is assumed to be normally distributed and independent of *b*_*i*_.

In our study design, we consider three time points at which the outcomes of interest are measured: t = 0, *T*_1*i*_ and *T*_2*i*_, where *T*_1*i*_ and *T*_2*i*_ are the respective time points at which individual *i* completes the first- and second-stage interventions. Therefore, *A*_1*i*_(*t*) takes the value of *A*_1*i*_ at times *T*_1*i*_ and *T*_2*i*_ and is equal to 0 at *t* = 0, and *A*_2*i*_(*t*) takes the value of *A*_2*i*_ at *T*_2*i*_ and is equal to 0 at time points 0, and *T*_1*i*_. In this way, *η*(*t*) and *β*(*t*) are piecewise linear fixed coefficients; therefore, model (1) at the three time points is equivalent to the following three linear mixed-effects submodels:

2 $$ \begin{array}{c}{Y}_{0i}={Y}_i(0)={m}_i(0)+{\varepsilon}_i(0)\\ {}={Z}_i\eta (0)+{X}_i^T(0)\beta (0)+{b}_i+{\varepsilon}_i(0)\\ {}={Z}_i{\eta}_0+{\beta}_{00}+{b}_i+{\varepsilon}_{0i}\end{array} $$

3 $$ \begin{array}{c}{Y}_{1i}=Y{\left({T}_{1i}\right)}_i={m}_i\left({T}_{1i}\right)+{\varepsilon}_i\left({T}_{1i}\right)\\ {}={Z}_i\eta \left({T}_{1i}\right)+{X}_i^T\left({T}_{1i}\right)\beta \left({T}_{1i}\right)+{b}_i+{\varepsilon}_i\left({T}_{1i}\right)\\ {}={Z}_i{\eta}_1+{\beta}_{01}+{\beta}_{11}{A}_{1i}+{\beta}_{31}{T}_{1i}+{b}_i+{\varepsilon}_{1i}\end{array} $$

and

4 $$ \begin{array}{c}{Y}_{2i}={Y}_i\left({T}_{2i}\right)={m}_i{T}_{2i}+{\varepsilon}_i\left({T}_{2i}\right)\\ {}={Z}_i\eta \left({T}_{2i}\right)+{X}_i^T\left({T}_{2i}\right)\beta \left({T}_{2i}\right)+{b}_i+{\varepsilon}_i\left({T}_{2i}\right)\\ {}={Z}_i{\eta}_2+{\beta}_{02}+{\beta}_{12}{A}_{1i}+{\beta}_2{A}_{2i}+{\beta}_{32}{T}_{2i}+{\beta}_4{A}_{1i}\cdot {A}_{2i}+{b}_i+{\varepsilon}_{2i}\\ {}={Z}_i{\eta}_2+{\beta}_{02}+{\beta}_{12}{A}_{1i}+{\beta}_{22}{A}_{2Ri}+{\beta}_{23}{A}_{2NRi}+{\beta}_{32}{T}_{2i}+{\beta}_{41}{A}_{1i}\cdot {A}_{2Ri}+{\beta}_{42}{A}_{1i}\cdot {A}_{2NRi}+{b}_i+{\varepsilon}_{2i}\end{array} $$

where in equations (2) through (4), *Y*_0*i*_, *Y*_1*i*_ and *Y*_2*i*_ are the outcome values at time 0, *T*_1*i*_ and *T*_2*i*_, respectively; *A*_1*i*_ is the indicator of the first-stage intervention options (−1 for M and +1 for B), *A*_2*i*_ = (*A*_2*Ri*_, *A*_2*NRi*_) is the indicator vector for the second-stage intervention options, where *A*_2*Ri*_ is the indicator for the second-stage intervention options for the responders to the first-stage intervention (1 = continue the initial intervention; −1 = reduce the intensity of the initial intervention) and *A*_2*NRi*_ is the indicator for the second-stage intervention options for the non-responders (1 = increase the initial intervention; −1 = augment the initial intervention with the other type of intervention), with *A*_2*Ri*_ =0 for non-responders and *A*_2*NRi*_ =0 for responders. *A*_1*i*_ ⋅ *A*_2*Ri*_ and *A*_1*i*_ ⋅ *A*_2*NRi*_ are the interaction effects of the first-stage intervention and second-stage intervention among responders and non-responders, respectively, in the submodel of *Y*_2*i*_ (i.e., submodel (4)).

Parameters *η*_0_, *η*_1_, *η*_2_ and *β*_00_, *β*_01_, *β*_02_ are the coefficients of the baseline covariates and intercepts at time points 0, *T*_1*i*_ and *T*_2*i*_, respectively; submodel (2) includes only baseline covariates as predictors for the outcomes at the beginning of the study (i.e., *Y*_0*i*_ at t = 0); submodel (3) models the outcome of interest at the intermediate time point of the study (i.e., *Y*_1*i*_ at t= *T*_1*i*_) and includes covariates *A*_1*i*_ and *T*_1*i*_, for which the corresponding coefficients *β*_11_ and *β*_31_ account for the direct effect of *A*_1*i*_ and indirect effects through *T*_1*i*_ on *Y*_1*i*_; submodel (4) includes all the main and interacting effects of the intervention options at each stage and the duration *T*_2*i*_ (*T*_2*i*_ = *T*_1*i*_ + *Δt*) as predictors, for which the coefficients *β*_12_ and *β*_32_ account for the delayed effect of *A*_1*i*_ and delayed indirect effects of *A*_1*i*_ through *T*_2*i*_. The coefficients *β*_2_ = (*β*_22_, *β*_23_) and *β*_4_ = (*β*_41_, *β*_42_) account for the effects of the second-stage interventions and the effects of their interactions with the first-stage interventions on the final outcome *Y*_2*i*_ (measured at the end of the study, *T*_2*i*_).

The conditional expectations for models (1)-(4) are provided in Additional file 2. We also provided conditional expectations of the final outcomes for each of the eight embedded adaptive interventions in the SMART design of Fig. 1 and four embedded adaptive interventions in the SMART design of Fig. 2 [see Additional file 2].

### Joint model

In addition to the TVMEM, we postulate a relative risk model for *T*_1*i*_ (time to the event of interest) as

5 $$ {h}_i(t)={h}_0(t) exp\left\{{\gamma}_1{A}_{1i}+{\gamma}_2{W}_i+\alpha {m}_i(0)\right\} $$

where *W*_*i*_ is a vector of the baseline covariates, which could be different from vector *Z*_*i*_ in model (1), and *h*_0_(⋅) is the baseline risk function. The underlying longitudinal measurement *m*_*i*_(0) at baseline (i.e., at time point t = 0), as approximated by the TVMEM, and at the first-stage intervention *A*_1*i*_ are included as predictors in model (5) because the time point at which an individual responds to the first-stage intervention (i.e., *T*_1*i*_) depends only on the type of first-stage intervention the subject received and the baseline characteristics.

We jointly estimate the coefficients in models (1) and (5) by using the maximum likelihood estimation method. To define the joint distribution of the time-to-event and longitudinal outcomes, we assume that the random effect *b*_*i*_ underlies both the longitudinal and survival processes for each subject. This means that the random effect accounts for both the association between the longitudinal and event outcomes and the correlation between the repeated measurements in the longitudinal process. We also assume that the longitudinal outcomes {*Y*_0*i*_, *Y*_1*i*_, *Y*_2*i*_} are independent of the time *T*_1*i*_ conditional on the random effect *b*_*i*_. Therefore, the joint likelihood contribution for the *ith* subject can be formulated as *p*(*T*_1*i*_, *δ*_*i*_, *Y*_*i*_; *θ*) =

$$ {\displaystyle \int p\left({T}_{1i},{\delta}_i\Big|{b}_i;\beta, \gamma, \alpha, \eta \right)}\left[{\displaystyle \prod_jp}\left\{{Y}_i\left({t}_{ij}\right)\Big|{b}_i;\beta, \eta \right\}\right]p\left({b}_i;{\sigma}_b\right)db $$, where *p*{*Y*_*i*_(*t*_*ij*_)|*b*_*i*_; *β*, *η*} is the univariate normal density for the longitudinal responses at time point *t*_*ij*_, which is the element from the vector *t*_*i*_ = {*t*_*si*_}_*s* = 0_^2^ = {0, *T*_1*i*_, *T*_2*i*_}; *p*(*b*_*i*_; *σ*_*b*_) is the normal density with standard deviation *σ*_*b*_ for the random effects *b*_*i*_; and *p*(*T*_1*i*_, *δ*_*i*_|*b*_*i*_; *β*, *γ*, *α*, *η*) is the likelihood for the time to the intermediate outcome and can be written as *p*(*T*_1*i*_, *δ*_*i*_|*b*_*i*_; *β*, *γ*, *α*, *η*) = $$ {\left\{{h}_i\left({T}_{1i}\Big|{m}_i(0);\beta, \gamma, \alpha, \eta \right)\right\}}^{\delta_i}\cdot $$*S*_*i*_(*T*_1*i*_|*m*_*i*_(0), *A*_1*i*_; *β*, *γ*, *α*, *η*) = $$ {\left\{{h}_i\left({T}_{1i}\Big|{m}_i(0);\beta, \gamma, \alpha, \eta \right)\right\}}^{\delta_i}\cdot $$$$ \exp \left\{-{\displaystyle {\int}_0^{T_{1i}}{h}_i\left(s\Big|{m}_i(0);\beta, \gamma, \alpha, \eta \right)}ds\right\} $$, where *δ*_*i*_ = *I*(*T*_1*i*_ < *t*_10_). Parameters in the model are estimated by maximizing the corresponding log-likelihood function with respect to (*β*, *γ*, *α*, *η*). We obtained the maximum likelihood estimates using the R package “JM” [30].

The parameters (*β*_12_, *β*_22_, *β*_23_, *β*_32_, *β*_41_, *β*_42_) in submodel (4) (i.e., the model of final outcome *Y*_2_) are of primary interest and were estimated using the two approaches described above.

The data organization and implementation of these methods is presented in Additional file 3.

### Simulations

For the example illustrated in Fig. 1, we considered two simulation scenarios in which *Y*_0_ and *Y*_1_ were simulated using submodels (2) and (3), respectively, and *Y*_2_ was simulated with and without the interaction terms (*A*_1*i*_ ⋅ *A*_2*Ri*_ and *A*_1*i*_ ⋅ *A*_2*NRi*_) in submodel (4). In both scenarios, we simulated 500 replicates of *n* = 1000 individuals, and randomly assigned subjects (with probability .5) to one of the two first-stage interventions (i.e., *A*_1_ to be equal to 1 [behavioral intervention] or −1 [medication]). Responders and non-responders to the initial interventions were then re-randomized (with probability .5) to one of the corresponding second-stage intervention options (i.e., *A*_2*R*_ and *A*_2*NR*_ were randomly assigned to be 1 or −1 and *A*_2*R*_ =0 for non-responders and *A*_2*NR*_ =0 for responders; see Fig. 1). In both scenarios, the random effects {*b*_*i*_}_*i* = 1_^*n*^ for subjects *i* = 1, 2, …, *n* were generated from the normal distribution with a mean of 0 and a standard deviation of 5, and baseline outcomes {*Y*_0*i*_}_*i* = 1_^*n*^ were simulated using submodel (2) with parameters *β*_00_ = 10 and *ε*_0*i*_ ~ *N*(0, 4^2^). The intermediate outcomes {*Y*_1*i*_}_*i* = 1_^*n*^ were simulated using submodel (3) with parameters *β*_01_ = 1, *β*_11_ = 0.2, and *β*_31_ = 0.1 in the first scenario; whereas outcomes {*Y*_1*i*_}_*i* = 1_^*n*^ in the second scenario were simulated with *β*_01_ = 1, *β*_11_ = 0.6, and *β*_31_ = 0.1, with a standard deviation of 5 (i.e., *ε*_1*i*_ ~ *N*(0, 5^2^) in both scenarios and satisfying the conditions *Y*_0*i*_ − *Y*_1*i*_ ≥ 9 (C = 9) if subject *i* is a responder and *Y*_0*i*_ − *Y*_1*i*_ < 9 if subject *i*(*i* = 1, 2, … *n*) is a non-responder.

The time points *T*_1*i*_ were generated from a left-truncated Weibull distribution (truncated from *t*_00_ =0.1, the start time for monitoring), with shape = 1 and scale= *exp*{*γ*_0_ + *γ*_1_*A*_1*i*_ + *αm*_*i*_(0)}, where *γ*_0_ = − 1.5, *γ*_1_ = 0.4, and *α* = 0.25, and those for whom *T*_1*i*_ was greater than 1 (non-responders), were assigned *T*_1*i*_ = *t*_10_ = 1 (the maximum time the first-stage intervention is administered [*t*_10_]). The indicator of response status was then defined by *δ*_*i*_ = *I*(*T*_1*i*_ < 1). The final outcomes *Y*_2*i*_(*i* = 1, …, *n*) were generated using submodel (4), with *ε*_2*i*_ ~ *N*(0, 5^2^). The values of the other parameters in submodel (4) are reported in Table 1 (without interactions) and Table 3 (with interactions).

**Table 1:** Simulation results for the design in Fig. 1: the estimated means, based on 500 replicates, are reported for coefficients in model (4)

Parameter estimation								
		*β*_12_	*β*_22_	*β*_23_	*β*_32_			
		(first-stage interventions *A*_1_)	(second-stage interventions for responders *A*_2*R*_)	(second-stage interventions for non-responders *A*_2*NR*_)	(time of intervention *T*_2_)			
True value		0.4	0.5	0.5	2			
Joint Model	Estimate	0.407	0.503	0.502	1.790			
	MSE	0.011	0.029	0.016	0.147			
	CI%	97.8 %	95.0 %	96.8 %	94.2 %			
	Length of CI	0.478	0.674	0.538	1.447			
TVMEM	Estimate	0.275	0.503	0.501	4.073			
	MSE	0.026	0.030	0.017	4.400			
	CI%	88.0 %	95.6 %	97.0 %	0.0 %			
	Length of CI	0.484	0.695	0.549	1.436			

CI%: Coverage probability of the 95 % confidence interval

*MSE* mean squared error

**Table 2:** Simulation results for the design in Fig. 1: the estimated means, based on 500 replicates, are reported for the final outcomes of the eight adaptive interventions embedded in the design

Mean of the final outcomes								
	(−1,-1,-1)	(−1,-1,1)	(1,-1,-1)	(1,-1,1)	(−1,1,-1)	(−1,1,1)	(1,1,-1)	(1,1,1)
Simulated means	4.538	5.087	5.554	6.275	4.988	5.536	5.842	6.564
Estimated means by Joint model	4.543	5.093	5.549	6.269	4.982	5.531	5.849	6.569
Estimated means by TVMEM	4.543	5.093	5.549	6.269	4.982	5.531	5.849	6.569

For the intervention strategy depicted in Fig. 1, there are eight adaptive interventions imbedded in the design and represented by the three indicators *A*_1_, *A*_2*R*_, and *A*_2*NR*_. For example, in adaptive intervention (*A*_1_, *A*_2*R*_, *A*_2*NR*_) = (−1, 1, 1), participants are initially randomized to the medication (*A*_1_ = − 1); those who respond are re-randomized to continue on the medication (*A*_2*R*_ = 1) and those who do not respond are re-randomized to increased medication (*A*_2*NR*_ = 1). Another example of an adaptive intervention is (*A*_1_, *A*_2*R*_, *A*_2*NR*_) = (1, 1, −1), in which participants are initially randomized to a behavioral intervention (*A*_1_ = 1); those who respond are re-randomized to continue on the behavioral intervention (*A*_2*R*_ = 1), and those who do not respond are re-randomized to an augmented arm (M + B, *A*_2*NR*_ = − 1).

For the design in Fig. 2, only the non-responders are re-randomized in the second stage. Therefore, there are four embedded adaptive interventions in this design, which are represented by the vector of two indicators (*A*_1_, *A*_2*NR*_). For example (−1,−1) represents the adaptive intervention in which participants are initially randomized to medication (*A*_1_ = − 1) and those who do not respond are re-randomized to the augmented arm (M + B, *A*_2*NR*_ = − 1), whereas responders continue on the medication arm.

Using this design, we also simulated the treatment of 1000 subjects. However, instead of using equal probability allocations as in Fig. 1, we used unequal probability allocations at both stages. Specifically, each of the 1000 subjects were initially assigned to either *A*_1_ = − 1 (medication) or *A*_1_ =1 (behavioral intervention) with probabilities 0.4 and 0.6, respectively. Then, the non-responders were re-allocated into either *A*_2*NR*_ = − 1 (augmented first-stage intervention, M + B) or *A*_2*NR*_ =1 (intensified first-stage intervention, M+ or B+) with probabilities 0.55 and 0.45, respectively; whereas all responders were continued on their initial interventions (therefore, *A*_2*R*_ =0) in their second stage. Random effects (*b*_*i*_), errors (*ε*_*i*_), and longitudinal outcomes (*Y*_0*i*_, *Y*_1*i*_(*i* = 1, …, *n*)) were generated as described for Fig. 1. The final outcomes, *Y*_2*i*_(*i* = 1, …, *n*), were also generated using submodel (4), but without the variable *A*_2*Ri*_, with the parameter values reported in Tables 5 and 7 for the two scenarios, respectively. In the first scenario, outcomes *Y*_2*i*_(*i* = 1, …, *n*) were simulated without interaction terms and with the parameter values shown in Table 5; in the second scenario, outcomes *Y*_2*i*_(*i* = 1, …, *n*) were simulated with interaction terms and with the parameter values shown in Table 7.

**Table 3:** Simulation results for the design in Fig. 1: the estimated means, based on 500 replicates, are reported for coefficients in model (4) with interactions

Parameter estimation								
		*β*_12_	*β*_22_	*β*_23_	*β*_32_	*β*_41_	*β*_42_	
		(first-stage interventions *A*_1_)	(second-stage interventions for responders *A*_2*R*_)	(second-stage interventions for non-responders *A*_2*NR*_)	(time of intervention *T*_2_)	(interaction term *A*_1_. *A*_2*R*_)	(interaction term *A*_1_. *A*_2*NR*_)	
True value		−0.4	0.5	0.4	2.0	0.55	−0.40	
Joint model	Estimate	−0.381	0.490	0.389	1.626	0.542	−0.397	
	MSE	0.014	0.038	0.019	0.305	0.037	0.019	
	CI%	97.4 %	95.6 %	96.8 %	87.0 %	96.8 %	98.0 %	
	Length of CI	0.521	0.789	0.593	1.671	0.789	0.593	
TVMEM	Estimate	−0.530	0.489	0.390	4.122	0.542	−0.396	
	MSE	0.029	0.039	0.020	4.697	0.037	0.020	
	CI%	88.2 %	95.8 %	96.4 %	0.0 %	97.0 %	97.8 %	
	Length of CI	0.525	0.809	0.605	1.618	0.809	0.605	

CI%: Coverage probability of the 95 % confidence interval

*MSE* mean squared error

**Table 4:** Simulation results for the design in Fig. 1: the estimated means, based on 500 replicates, are reported for the final outcomes of the eight adaptive interventions embedded in the design with interactions in model (4)

Mean of the final outcomes								
	(−1,-1,-1)	(−1,-1,1)	(1,-1,-1)	(1,-1,1)	(−1,1,-1)	(−1,1,1)	(1,1,-1)	(1,1,1)
Simulated means	5.456	6.306	5.009	5.000	5.400	6.249	5.577	5.565
Estimated means by Joint model	5.456	6.306	5.009	5.000	5.400	6.249	5.577	5.565
Estimated means by TVMEM	5.456	6.306	5.009	5.000	5.400	6.249	5.577	5.565

### An alternate simulation approach

For the design illustrated in Fig. 1, we performed an alternate simulation approach that does not simulate values for *T*_1*i*_ from the Weibull distribution. Instead, we considered a situation in which values of *Y*_1*i*_ are monitored and *T*_1*i*_ is the value for which the *Y*_1*i*_ crosses the pre-specified boundary condition for the first time. In this simulation approach, random effects {*b*_*i*_}_*i* = 1_^*n*^ and error terms *ε*_0_, *ε*_1_ and *ε*_2_ were all simulated the same way as described above. Baseline outcomes {*Y*_0*i*_}_*i* = 1_^*n*^ are simulated using submodel (2) with *β*_00_ = 2 and *ε*_0*i*_ ~ *N* (0, 2^2^). Furthermore, we defined an individual *i,* as a responder if he/she had a certain percentage reduction in the intermediate outcome value, *Y*_1*i*_, compared to his/her baseline value *Y*_0*i*_. This may be a more appropriate definition of responders in some practical scenarios than a simple reduction by a fixed amount (e.g., C = 9) as was used in the previous simulations. In this simulation, those with a 40 % reduction from their baseline values were considered responders. The parameter values used for submodel (3) were *β*_01_ = −2, *β*_11_ = −0.5, and *β*_31_ = 5 . For an individual *i*, we first simulated *ε*_1*i*_ ~*N* (0, 2^2^), and calculated *T*_1i_^*^ for which the *β*_01_ + *β*_11_*A*_1*i*_ + *β*_31_*T*_1i_^*^ + *b*_*i*_ + *ε*_1*i*_ equals the 40 % reduction from *Y*_0*i*_, the baseline value. Therefore, we define *T*_1*i*_ = *t*_00_, if *T*_1i_^*^ < *t*_00_; *T*_1*i*_ = *T*_1i_^*^, if *t*_00_ ≤ *T*_1i_^*^ ≤ *t*_10_; and *T*_1*i*_ = *t*_00_, if *T*_1i_^*^ ≥ *t*_10_. Then, *T*_1*i*_ is substituted in the right side of equation (3) to obtain the value of *Y*_1*i*_ for the individual *i*(*i* = 1, …, *n*). The final outcomes *Y*_2*i*_(*i* = 1, …, *n*) were generated using submodel (4), with *ε*_2*i*_ ~ *N* (0, 2^2^). As previously, we simulated 500 replicates of n = 1000 individuals in each trial, and randomly assigned subjects (with probability 0.5) to one of the two first-stage interventions (i.e., *A*_1_ to be equal to 1 [behavioral intervention] or −1 [medication]). Responders and non-responders to the initial interventions were then re-randomized (with probability 0.5) to one of the corresponding second-stage intervention options (i.e., *A*_2*R*_ and *A*_2*NR*_ were randomly assigned to be 1 or −1 and *A*_2*R*_ =0 for non-responders and *A*_2*NR*_ =0 for responders; see Fig. 1).

We evaluated the performance of our two proposed analytic approaches in these simulated data sets by measuring the (a) means of the estimates of each of the adaptive interventions embedded in the design, (b) parameter estimates in the model, (c) mean squared error (MSE), (d) estimated coverage probability of the 95 % confidence interval, and (e) length of the confidence interval.

Using these simulations parameters, we simulated two trials with identical sample sizes: (a) the time-varying SMART design and (b) the standard SMART design. We evaluated the performance of the time-varying SMART design and an analogous standard SMART design by measuring the (a) power to select the optimal embedded intervention, and (b) associated cost.

## Results

Tables 1-4 show the results of the two simulation scenarios based on the design shown in Fig. 1. Similarly, Tables 5-8 show the results of the two simulation scenarios for the design in Fig. 2.

In Table 1 the true parameters were the coefficient of the first-stage interventions, *β*_12_ = 0.4; coefficient of the second-stage intervention for responders, *β*_22_ = 0.5; coefficient of the second-stage intervention for non-responders, *β*_23_ = 0.5; and coefficient of T_2_, the total time of the first- and second-stage interventions, *β*_32_ = 2. The estimates obtained using TVMEM were ${\widehat{\beta}}_{12}=0.275,\kern0.5em {\widehat{\beta}}_{22}=0.503,\kern1em {\widehat{\beta}}_{23}=0.501,$ and ${\widehat{\beta}}_{32}=4.073$, while the estimates obtained using the joint model were ${\tilde{\beta}}_{12}=0.407,\kern0.5em {\tilde{\beta}}_{22}=0.503,\kern0.5em {\tilde{\beta}}_{23}=0.502,$ and ${\tilde{\beta}}_{32}=1.790$. Both approaches estimated coefficients *β*_22_ and *β*_23_ accurately. The parameters *β*_12_ and *β*_32_ were estimated accurately using the joint model, but poorly using the TVMEM. Similarly, in terms of the MSE, the length of the 95 % confidence interval, and the estimated coverage probability of the 95 % confidence interval, both approaches performed similarly for estimating *β*_22_ and *β*_23_, but joint modeling performed better for estimating *β*_12_ and *β*_32_. For example, for *β*_12_, the estimated coverage probability obtained using the TVMEM was 88 %; whereas that obtained from the joint model was 97.8 %.

For each of the eight embedded adaptive interventions in the design, Table 2 shows that both approaches accurately estimated the means of the final outcome, *E*[*Y*_2_|(*A*_1_, *A*_2*R*_, *A*_2*NR*_)]. For example, the simulated means of the adaptive interventions (*A*_1_, *A*_2*R*_, *A*_2*NR*_) = (−1, −1, − 1), (−1, 1, 1), and (1, 1, 1) were 4.538, 5.536, and 6.564, respectively, and the estimated means were 4.543, 5.531, and 6.569, respectively, using the TVMEM and joint model.

Tables 3 and 4 show results similar to those in Tables 1 and 2, respectively. In Table 3, the coefficient of interaction of the first-stage interventions and second-stage interventions among responders is denoted by *β*_41_, and the coefficient of interaction of the first-stage interventions and second-stage interventions among non-responders is denoted by *β*_42_. As shown in Table 3, both TVMEM and joint modeling accurately estimated parameters *β*_22_, *β*_23_, *β*_41_, and *β*_42_, with little difference in the MSE, estimated coverage probability, and length of the 95 % confidence interval. However, as in Table 1, the joint modeling approach estimated *β*_12_ and *β*_32_ more accurately than the TVMEM approach. For example, the true coefficient of *T*_2_ was *β*_32_ = 2.0, which was poorly estimated as 4.122 using the TVMEM and estimated as 1.626 using the joint model. Table 4 shows that the estimated means of the eight adaptive interventions obtained from both analytical approaches were identical and close to the simulated means up to the third decimal.

Similar trends were observed in Tables 5-8 for the two simulations of Fig. 2. *β*_12_ and *β*_32_ were better estimated using the joint modeling approach, whereas all the other parameters and the means of the final outcomes of the four adaptive interventions embedded in the design were accurately estimated using both approaches.

In Table 5, the true coefficient values of *β*_12_ =0.450 and *β*_32_ =2.0 were estimated as ${\widehat{\beta}}_{12}=0.388$ and ${\widehat{\beta}}_{32}=4.046$ using the TVMEM, and as ${\tilde{\upbeta}}_{12}=0.456$ and ${\tilde{\upbeta}}_{32}=1.767$ using the joint model. Coefficient *β*_23_ was accurately estimated using both models. As for the four adaptive interventions (i.e. (*A*_1_, *A*_2*NR*_) = (−1,1), (−1,-1), (1, 1) and (1, −1)) embedded in the design of Fig. 2, Table 6 shows that the simulated means were 5.213, 4.802, 6.330, and 5.805, respectively, and the estimated means were 5.228, 4.790, 6.344, and 5.793, respectively, using the TVMEM, and 5.230, 4.788, 6.345, and 5.792, respectively, using the joint model.

**Table 5:** Simulation results for the design in Fig. 2: the estimated means, based on 500 replicates, are reported for coefficients in model (4)

Parameter estimation				
		*β*_*12*_	*β*_*23*_	*β*_*32*_
		(first-stage interventions *A*_1_)	(second-stage interventions for non-responders *A*_2*NR*_)	(time of intervention *T*_2_)
True value		0.450	0.40	2.0
Joint model	Estimate	0.456	0.441	1.767
	MSE	0.013	0.017	0.168
	CI%	95.6 %	95.6 %	93.6 %
	Length of CI	0.482	0.536	1.452
TVMEM	Estimate	0.388	0.439	4.046
	MSE	0.016	0.017	4.297
	CI%	94.0 %	96.0 %	0.0 %
	Length of CI	0.489	0.547	1.442

CI%: Coverage probability of the 95 % confidence interval

*MSE* mean squared error

**Table 6:** Simulation results for the design in Fig. 2: the estimated means, based on 500 replicates, are reported for the final outcomes of the four adaptive interventions embedded in the design

Mean of the final outcomes				
	(−1,1)	(−1,-1)	(1,1)	(1,-1)
Simulated means	5.213	4.802	6.330	5.805
Estimated means by joint model	5.230	4.788	6.345	5.792
Estimated means by TVMEM	5.228	4.790	6.344	5.793

In Table 7 shows that the true parameters *β*_12_ =0.40 and *β*_32_ =2.0 were respectively estimated as ${\widehat{\upbeta}}_{12}$ =0.298 and ${\widehat{\beta}}_{32}$ =4.308 using the TVMEM, and as ${\tilde{\upbeta}}_{12}$ =0.408 and ${\tilde{\upbeta}}_{32}$ =1.784 using the joint model. The other two parameters, *β*_23_ and *β*_42_, were accurately estimated using both approaches. Table 8 shows that the means were accurately estimated using both approaches.

**Table 7:** Simulation results for the design in Fig. 2: the estimated means, based on 500 replicates, are reported for coefficients in model (4) with interactions

Parameter estimation					
		*β*_*12*_	*β*_*23*_	*β*_*32*_	*β*_*42*_
		(first-stage intervention *A*_1_)	(second-stage interventions for responder *A*_2*NR*_)	(time of intervention *T*_2_)	(interaction term*A*_1_ *A*_2*NR*_))
True value		0.4	0.4	2.0	−0.4
Joint model	Estimate	0.408	0.422	1.784	−0.400
	MSE	0.012	0.020	0.157	0.019
	CI%	98.2 %	97.2 %	95.6 %	97.2 %
	Length of CI	0.513	0.594	1.542	0.593
TVMEM	Estimate	0.298	0.419	4.308	−0.399
	MSE	0.021	0.021	5.439	0.020
	CI%	94.8 %	97.4 %	0.0 %	97.4 %
	Length of CI	0.520	0.608	1.532	0.607

CI%: Coverage probability of the 95 % confidence interval; MSE: mean squared error

**Table 8:** Simulation results for the design in Fig. 2: the estimated means, based on 500 replicates, are reported for the final outcomes of the four adaptive interventions embedded in the design with interactions in model (4)

Mean of the final outcomes					
	(−1,1)	(−1,-1)	(1,1)	(1,-1)	
Simulated means	5.487	4.622	6.032	6.059	
Estimated means by joint model	5.502	4.610	6.047	6.046	
Estimated means by TVMEM	5.500	4.611	6.045	6.048	

Tables 9 and 10 show the results from the alternative simulation strategy. In Table 9, the true coefficient values of *β*_12_ = −0.6 and *β*_32_ = −1.5 were estimated as ${\widehat{\upbeta}}_{12}$ = −0.534 and ${\widehat{\beta}}_{32}$ = −2.367 using the TVMEM, and as ${\tilde{\upbeta}}_{12}$ = −0.608 and ${\tilde{\upbeta}}_{32}$ = −1.338 using the joint model. Coefficients *β*_22_, *β*_23_ and the means of the final outcomes of the eight adaptive interventions embedded in the design were accurately estimated using both approaches (Table 10).

**Table 9:** Simulation results from the alternative simulation approach: the estimated means, based on 500 replicates, are reported for coefficients in model (4)

Parameter estimation								
		*β*_*12*_	*β*_*23*_	*β*_*32*_	*β*_*42*_			
		(first-stage intervention *A*_1_)	(second-stage interventions for responder *A*_2*NR*_)	(time of intervention *T*_2_)	(interaction term *A*_1_ . *A*_2*NR*_)			
True value		−0.6	0.5	0.4	−1.5			
Joint model	Estimate	−0.608	0.492	0.447	−1.338			
	MSE	0.002	0.003	0.018	0.034			
	CI%	97 %	96 %	87 %	70 %			
	Length of CI	0.199	0.192	0.376	0.411			
TVMEM	Estimate	−0.534	0.492	0.448	−2.367			
	MSE	0.007	0.003	0.020	0.763			
	CI%	74 %	93 %	84 %	0 %			
	Length of CI	0.197	0.191	0.376	0.529			

CI%: Coverage probability of the 95 % confidence interval

*MSE* mean squared error

### Comparison of power between the time-varying SMART design and the standard SMART design

**Fig. 3:**
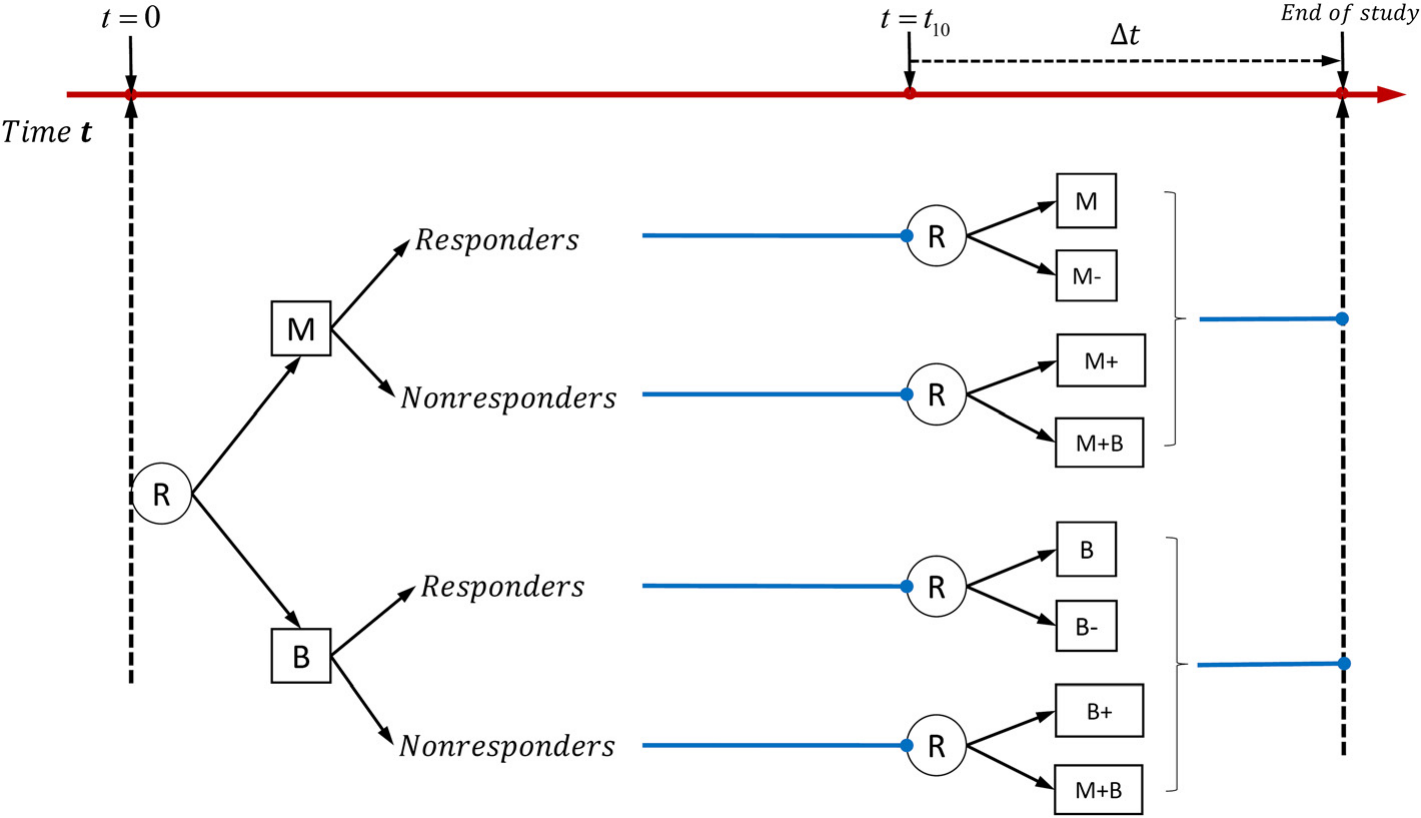
Example of standard SMART design with equal probability allocation: each participant is randomized twice

We analyzed the time-varying SMART design’s ability to select the most optimal embedded intervention and compared the associated power to that of the standard SMART design. We performed the comparison by conducing two trials with identical sample sizes and intervention effects using (a) the time-varying SMART design and (b) the standard SMART design. Figure 3 represents the standard SMART design that is analogous to the time-varying SMART design depicted in Fig. 1. The major difference between the two designs is that in the time-varying SMART design, a responder is re-randomized to the second-stage intervention at a random response time *T*_1_ (< *t*_10_); whereas in the standard SMART design, everyone is re-randomized at a fixed time point *t*_10_. Responders are defined similarly in both designs. In our example, a subject is considered a responder to the first-stage intervention if there is a significant decrease in the number of cigarettes the person smoked per day. The second-stage intervention is identical for both designs.

**Fig. 4:**
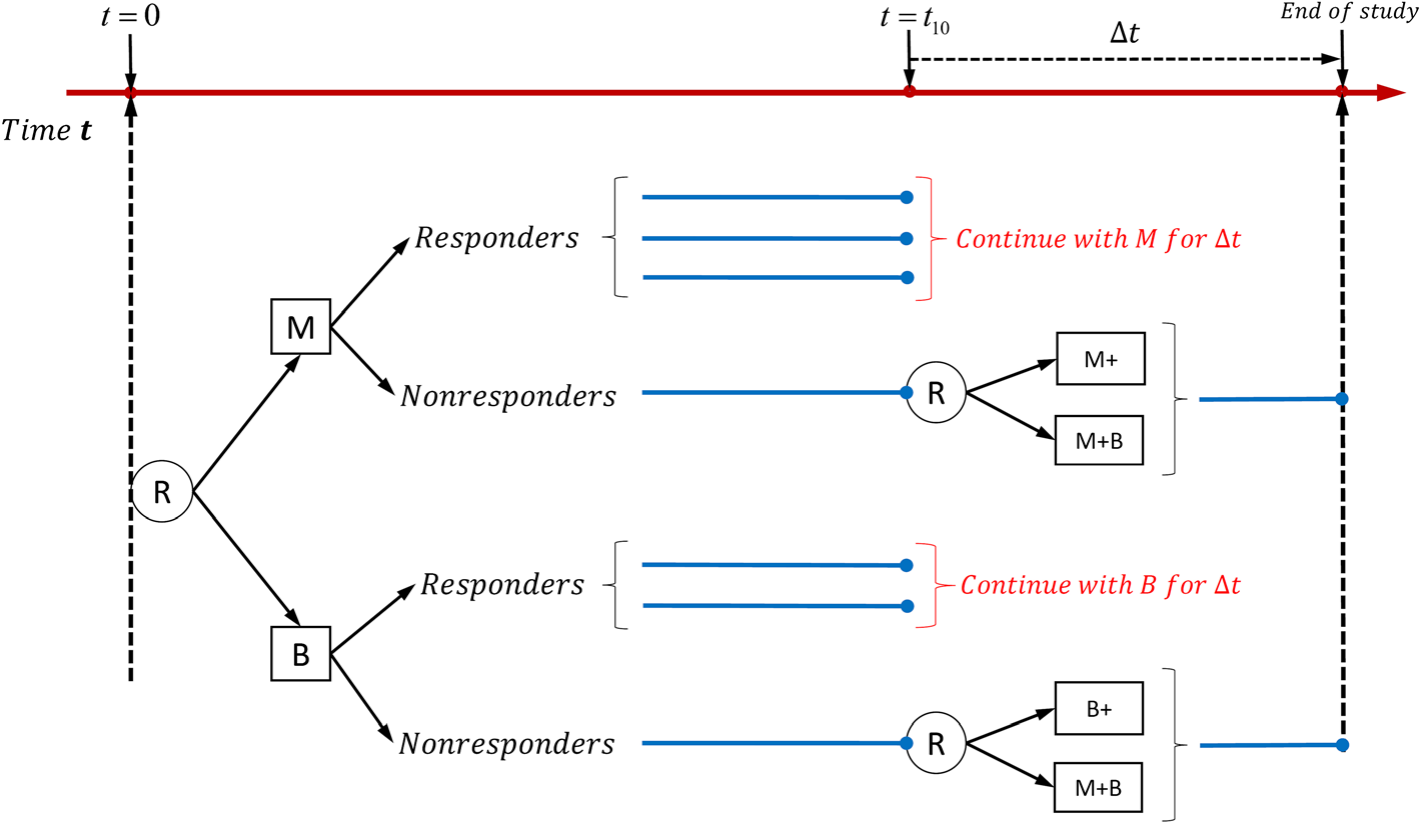
Example of standard SMART design: only non-responders are re-randomized in the second stage

For both designs, we calculated the percentage of times that the best embedded intervention is selected (i.e., the power of the design). We simulated six parameter scenarios: the true parameters for the coefficient of the first-stage interventions, *β*_21_; coefficient of the second-stage intervention for responders, *β*_22_; coefficient of the second-stage intervention for non-responders, *β*_23_; coefficient of *T*_2_, the total time of the first- and second-stage interventions, *β*_32_; coefficient of interaction of the first-stage interventions and second-stage interventions among responders *β*_41_; and the coefficient of interaction of the first-stage interventions and second-stage interventions among non-responders *β*_42_. The simulated values of each of these parameters are reported in Tables 11 and 12. The simulation results are based on 500 replicates and are shown in Table 11 for comparing the two designs in Fig. 1 (time-varying SMART) and Fig. 3 (analogous standard SMART). Overall, both designs were equally effective in selecting the optimal embedded adaptive intervention. For example, when *β*_21_ = 0.4, *β*_22_ = 0.5, *β*_23_ = 0.5 and *β*_32_ = 2, using the joint model and implementing the time-varying SMART design showed 82.8 % power to select the optimal embedded adaptive intervention; whereas the power associated with the standard SMART design was 83.0 %. Similar results were obtained when comparing the time-varying SMART design in Fig. 2 and the standard SMART design in Fig. 4 (see Table 12).

**Table 10:** Simulation results from the alternative simulation approach: the estimated means, based on 500 replicates, are reported for the final outcomes of the eight adaptive interventions embedded in the design

Mean of the final outcomes								
	(−1,-1,-1)	(−1,-1,1)	(1,-1,-1)	(1,-1,1)	(−1,1,-1)	(−1,1,1)	(1,1,-1)	(1,1,1)
Simulated means	−0.208	−0.070	−1.589	−1.356	0.678	0.810	−0.918	−0.689
Estimated means by Joint model	−0.203	−0.066	−1.595	−1.359	0.675	0.804	−0.915	−0.683
Estimated means by TVMEM	−0.203	−0.063	−1.594	−1.363	0.672	0.804	−0.912	−0.684

**Table 11:** Power to select the optimal embedded adaptive intervention strategy for designs in Figs. 1 and 3

Comparison of designs in Figs. 1 and 3	*β*_*12*_	*β*_*22*_	*β*_*23*_	*β*_*32*_	*β*_*41*_	*β*_*42*_	Power to select optimal embedded adaptive strategy	
							Time-varying SMART	Standard SMART
Without interaction	0.4	0.5	0.5	2			82.8 %	83.0 %
	0.3	−0.2	0.4	2			60.2 %	59.2 %
	0.3	−0.5	0.4	2			76.2 %	75.0 %
With interaction	0.4	0.5	0.5	2	0.5	−0.3	99.2 %	97.0 %
	0.6	0.5	0.4	2	0.2	0.2	63 %	62.8 %
	0.6	−0.5	−0.5	2	0.2	−0.3	72.2 %	73.4 %

**Table 12:** Power to select the optimal embedded adaptive intervention strategy for designs in Figs. 2 and 4

Comparison of designs in Figs. 2 and 4.	*β*_*12*_	*β*_*23*_	*β*_*32*_	*β*_*42*_	Power to select optimal embedded adaptive strategy	
					Time-Varying SMART	Standard SMART
Without interaction	0.5	0.5	2		92.8 %	90.6 %
	0.45	0.4	2		86.6 %	83.6 %
	−0.2	0.2	2		68.4 %	66.2 %
With interaction	0.4	0.4	2	−0.4	98.2 %	97.4 %
	0.2	0.2	2	−0.4	88.4 %	87.6 %
	0.4	0.1	2	−0.25	77.4 %	78.6 %

### Comparison of the cost associated with conducting the time-varying SMART design versus that associated with conducting the standard SMART design

To assess the cost associated with the conducting trials using these two competing designs, we considered a linear cost function for both SMART designs. Let *c*_1_ and *c*_2_ be the cost of the medication (M) and behavioral intervention (B), respectively. Additionally, we assumed that the reduced and increased intensity of the first-stage intervention are at half and twice the cost of the first-stage intervention, respectively, and that augmentation of the first-stage intervention in the second stage (M + B) has the cost *c*_1_ + *c*_2_. Using these parameters, the cost for the time-varying SMART design in Fig. 1 is

$$ \begin{array}{c} cost={c}_1\left({\displaystyle \sum_{A_{1i}=M}{T}_{1i}}\right)+{c}_1\left({\displaystyle \sum_{A_{2i}=\mathrm{M}}\varDelta \mathrm{t}}\right)+\left(\frac{c_1}{2}\right)\left({\displaystyle \sum_{A_{2i}=M-}\varDelta \mathrm{t}}\right)+\left(2{c}_1\right)\left({\displaystyle \sum_{A_{2i}=M+}\varDelta \mathrm{t}}\right)+\\ {}\kern5em {c}_2\left({\displaystyle \sum_{A_{1i}=B}{T}_{1i}}\right)+{c}_2\left({\displaystyle \sum_{A_{2i}=\mathrm{B}}\varDelta \mathrm{t}}\right)+\left(\frac{c_2}{2}\right)\left({\displaystyle \sum_{A_{2i}=B-}\varDelta \mathrm{t}}\right)+\left(2{c}_2\right)\left({\displaystyle \sum_{A_{2i}=B+}\varDelta \mathrm{t}}\right)+\left({c}_1+{c}_2\right)\left({\displaystyle \sum_{A_{2i}=M+B}\varDelta \mathrm{t}}\right),\end{array} $$

and the cost for the corresponding standard SMART in Fig. 3 is

$$ \begin{array}{l} cost={c}_1\left({\displaystyle \sum_{A_{1i}=M}{t}_{10}}\right)+{c}_1\left({\displaystyle \sum_{A_{2i}=M}\varDelta \mathrm{t}}\right)+\left(\frac{c_1}{2}\right)\left({\displaystyle \sum_{A_{2i}=M-}\varDelta \mathrm{t}}\right)+\left(2{c}_1\right)\left({\displaystyle \sum_{A_{2i}=M+}\varDelta \mathrm{t}}\right)+\\ {}\kern5em {c}_2\left({\displaystyle \sum_{A_{1i}=B}{t}_{10}}\right)+{c}_2\left({\displaystyle \sum_{A_{2i}=B}\varDelta \mathrm{t}}\right)+\left(\frac{c_2}{2}\right)\left({\displaystyle \sum_{A_{2i}=B-}\varDelta \mathrm{t}}\right)+\left(2{c}_2\right)\left({\displaystyle \sum_{A_{2i}=B+}\varDelta \mathrm{t}}\right)+\left({c}_1+{c}_2\right)\left({\displaystyle \sum_{A_{2i}=M+B}\varDelta \mathrm{t}}\right).\end{array} $$

Similarly, the cost for the time-varying SMART in Fig. 2 is

$$ \begin{array}{c} \cos t={c}_1\left({\displaystyle \sum_{A_{1i}=M}{T}_{1i}}\right)+{c}_1\left({\displaystyle \sum_{A_{2i}=M}\varDelta \mathrm{t}}\right)+\left(2{c}_1\right)\left({\displaystyle \sum_{A_{2i}=M+}\varDelta \mathrm{t}}\right)+\\ {}\kern5em {c}_2\left({\displaystyle \sum_{A_{1i}=B}{T}_{1i}}\right)+{c}_2\left({\displaystyle \sum_{A_{2i}=B}\varDelta \mathrm{t}}\right)+\left(2{c}_2\right)\left({\displaystyle \sum_{A_{2i}=B+}\varDelta \mathrm{t}}\right)+\left({c}_1+{c}_2\right)\left({\displaystyle \sum_{A_{2i}=M+B}\varDelta \mathrm{t}}\right),\end{array} $$

and the cost for the corresponding standard SMART in Fig. 4 is

$$ \begin{array}{l} cost={c}_1\left({\displaystyle \sum_{A_{1i}=M}{t}_{10}}\right)+{c}_1\left({\displaystyle \sum_{A_{2i}=M}\varDelta \mathrm{t}}\right)+\left(2{c}_1\right)\left({\displaystyle \sum_{A_{2i}=M+}\varDelta \mathrm{t}}\right)+\\ {}\kern4em {c}_2\left({\displaystyle \sum_{A_{1i}=B}{t}_{10}}\right)+{c}_2\left({\displaystyle \sum_{A_{2i}=B}\varDelta \mathrm{t}}\right)+\left(2{c}_2\right)\left({\displaystyle \sum_{A_{2i}=B+}\varDelta \mathrm{t}}\right)+\left({c}_1+{c}_2\right)\left({\displaystyle \sum_{A_{2i}=M+B}\varDelta \mathrm{t}}\right).\end{array} $$

Note that in the above equations, *T*_1*i*_ = t_10_ for non-responders, and $\left({c}_1+{c}_2\right)\left({\displaystyle \sum_{A_{2i}=M+B}\varDelta \mathrm{t}}\right)$ is the cost of the second stage for all the subjects assigned to the intervention M + B.

**Fig. 5:**
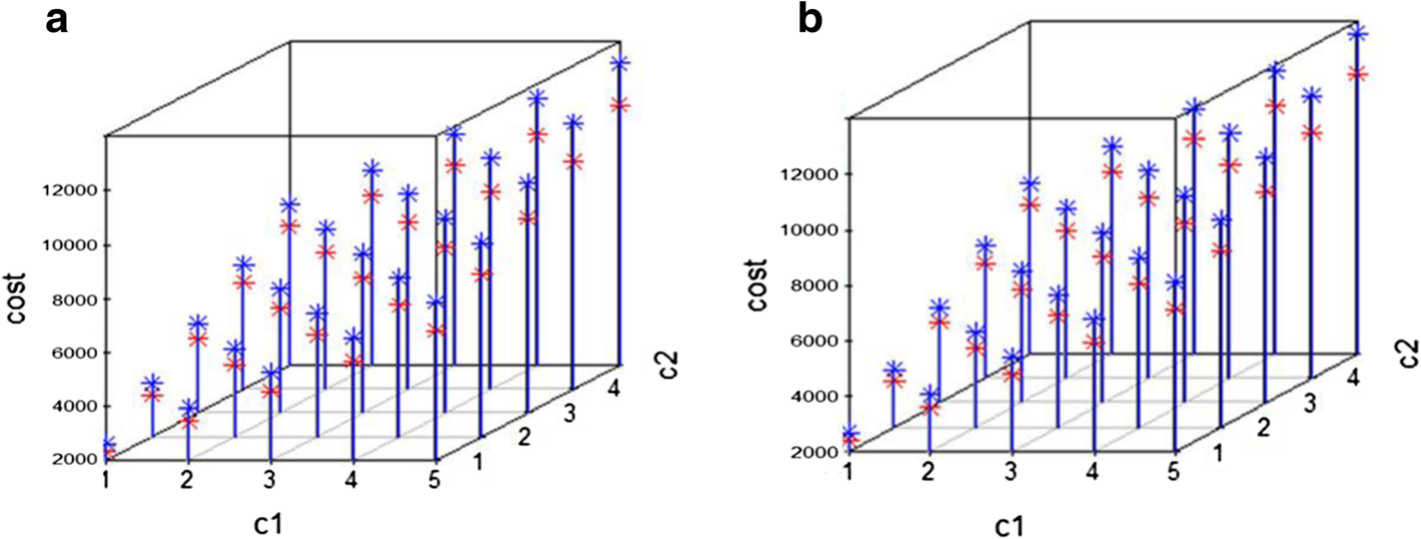
The cost associated with implementing a standard SMART (blue) and equivalent time-varying SMART (red)

Figure 5 shows the cost as a function of *c*_1_ and *c*_2_, where red represents the cost of the time-varying SMART design and blue represents the cost of the standard SMART design. We can see that the cost of the time-varying SMART is less than the cost of the standard SMART in all scenarios. Table 13 shows the average costs and standard deviations calculated at select values of *c*_1_ and *c*_2_ based on 1000 replicates. For example, when the unit costs are *c*_1_ = 2 and *c*_2_ = 1 for medication and behavioral intervention, the average cost of the time-varying SMART in Fig. 1 is 3446.5, with standard deviation 49.87, while the average cost of the corresponding standard SMART is 3935.8, with standard deviation 41.47. Thus, the cost of the standard SMART is about 12 % higher than that of the time-varying SMART in this scenario.

**Table 13:** Examples of the average cost for time-varying SMART and the standard SMART

	*c*_1_	*c*_2_	Average Cost(SD)	
			Time-varying SMART	Standard SMART
Design in Figs. 1 and 3: All the subjects are re-randomized	2	1	3446.5(49.87)	3935.8(41.47)
	1	1	2325.7(29.82)	2631.4(19.15)
	1	2	3526.5(57.00)	3953.2(46.63)
Design in Figs. 2 and 4: Only non-responders are re-randomized	2	1	3593.4(45.40)	4056.9(36.52)
	1	1	2416.1(24.12)	2704.9(14.59)
	1	2	3655.5(53.53)	4056.7(42.77)

## Discussion

In the standard SMART design, the timing of allocating the intervention is generally ignored, which leads to a model of regression without the predictor of a time variable. Therefore, in this article, we proposed a time-varying SMART design that allows the re-randomization to the second-stage interventions to occur at different time points for different individuals. The two modeling approaches we proposed for analyzing data using such time-varying SMART designs provided good estimations of the means of the final outcomes of all the embedded interventions. However, the joint modeling approach provided more accurate parameter estimates and higher estimated coverage probability than the TVMEM, and thus we recommend the joint model for analyzing data generated from time-varying SMART designs.

In the examples illustrated in Figs. 1 and 2, a participant was defined as a responder if there was a significant decrease in the number of cigarettes the participant smoked per day. One may question the validity of re-randomizing individuals who have a quick response to the first-stage intervention because such a response indicates the effectiveness of the intervention. However, if significant adverse effects are associated with the intervention (e.g., radiation therapy for many types of cancer is commonly associated with skin damage [31], fatigue [32, 33], diarrhea [34, 35], and rectal bleeding [36]), it is reasonable to shorten the duration of the intervention to avoid side effects. Therefore, the allocation strategy for the responders in the examples of the time-varying SMART design makes it more efficient than the standard SMART design.

We proposed two approaches for analyzing the longitudinal outcomes obtained from the time-varying SMART design: the TVMEM and the joint model. According to the simulation results, the joint modeling approach better estimated the effects of the duration of the intervention (i.e., *T*_2_) and the first-stage interventions (i.e., *A*_1_) in model (4). More specifically, the joint modeling approach had more accurate estimates, smaller MSEs, higher estimated coverage probabilities, and smaller 95 % confidence intervals (i.e., smaller estimated standard deviations) for the coefficients of the effects of the first-stage intervention and the time of intervention. Because we wanted to illustrate the cost efficiency of the proposed time-varying SMART design and its ability to select the optimal embedded adaptive intervention, we implemented a rather simplified linear mixed-effects submodels (2)-(4) of the more general TVMEM in model (1). We showed that the joint model performs better than the TVMEM in analyzing the data collected from such time-varying SMART designs. The joint modeling approach extracts part of the information contained in the time of the response, which is a function of the first-stage treatment assignment. Also, the association between the longitudinal and event outcomes is accounted for by the random effect that underlies both the longitudinal and survival processes for each subject. Therefore, although complex, time-varying SMART designs may require more complicated models for time and an extra layer of joint modeling, and as such one would expect a better performance from joint modeling in general. Nevertheless, both modeling approaches performed well in estimating the other parameters and the mean of the final outcomes for each adaptive intervention embedded in the corresponding designs. Furthermore, equation (1) is a general form of TVMEM, and in our study is equivalent to equations (2) ~ (4) at time points *t* = 0, *T*_1*i*_, *T*_2*i*_ for each subject *i. T*_1*i*_ is a subject-specific random variable, and coefficients in equation (3) can also be subject-specific. However, in practice, modeling coefficients to be subject-specific may lead to the estimation of too many parameters which, in some scenarios, may not be identifiable, particularly with small sample sizes. Therefore, as an initial attempt, we modeled *T*_1*i*_ as a subject-specific random variable and the coefficients as fixed parameters. For example, coefficients *β*_0_(*t*), *β*_1_(*t*), *β*_3_(*t*) in equation (1) are fixed coefficients *β*_01_, *β*_11_, *β*_31_ in equation (3), as model (1) is equivalent to submodel (3) at time point *T*_1*i*_. More complicated models such as subject-specific and time-varying coefficients in submodels (2)-(4) can be considered, if the sample sizes are large.

We also illustrated the effectiveness of the joint modeling approach in accurately estimating the parameters even when no specific model was assumed for the duration of the first-stage intervention, *T*_1*i*_. The conclusions were qualitatively similar as that in the simulation where Weibull model was assumed for the duration of the first-stage intervention.

In the scenarios we considered here, the time at which individuals were re-randomized was assessed only for responders to the first-stage intervention. However, one may also consider varying times for the non-responders and for the second-stage interventions. For example, a non-responder showing severe side effects or no trend towards achieving intermediate goals may be re-randomized sooner than *t*_10_. The analytic approaches for such designs would be similar to the joint or time-varying mixed effects models proposed in this manuscript, for example, with an extra submodel for the duration of the second-stage interventions.

Instead of randomization with certain pre-defined probabilities (e.g., in the first two simulation scenarios, randomization with probability 0.5 was used for both stages; in the last two scenarios, unequal randomization with probabilities 0.4(0.6) and 0.55(0.45) was used for the two stages, respectively), information concerning potential moderators could be used to tailor and assign the interventions. For example, the choice of the first-stage intervention options could depend on the severity of the subject’s smoking habit at the beginning of the study; whereas the choice of the second-stage intervention option could depend on the subject’s adherence to the first-stage intervention. The analysis of such a randomization scheme would require assigning weights for each subject [37].

We also compared the cost and power associated with selecting the optimal embedded adaptive intervention for the proposed time-varying SMART design versus that for the analogous standard SMART design. Our simulation results showed similar power for the two designs. We used a linear cost function to assess the cost efficiency of the proposed design and found that it can have substantially lower cost than the standard design. Several other forms of cost functions can be used to assess cost efficiency. However, as long as the cost is an increasing function of time, the proposed time-varying SMART design will have lower cost than the standard SMART design. Therefore, the time-varying SMART design can be used to study how the intensity and combination of two types of interventions might be adapted to a subject’s progress in a cost- and time-efficient manner.

In our study, we assume that there is no unmeasured confounder. As suggested by Chakraborty and Murphy [38], the assumption of “no unmeasured confounders” holds in a SMART design if the randomization probabilities of A_1_ at most depend on the baseline covariates, and the randomization probabilities of A_2_ at most depend on the baseline covariates, the intermediate outcome, and A_1_. We performed additional simulations to investigate the role of unmeasured confounders on the parameter estimations. From these simulations, we see that when the unmeasured confounders affect only *T*_*1*_ and *Y*_*1*_, the parameter estimation is still accurate (Additional file 4: Table S4). However, when these unmeasured confounders affect *Y*_*2*_, there is bias in the estimation of *T*_*2*_ (Additional file 4: Tables S5-S6).

In the ADHD SMART study discussed by Nahum-Shani et al. [20], a weighted average was applied to the final outcomes when their primary goal of the study was to compare the imbedded adaptive intervention options in the SMART. In our Time-Varying SMART study, we used regression-based methods to identify more efficient adaptive decision rules for each subject along with their longitudinal outcomes. Similar to the analytic process of the standard SMART design by Q-learning in which a regression model for the outcome is postulated at each decision as a function of the patient’s information to that point, our TVMEM in equation (1) is equivalent to submodels (2)-(4) at three time points of longitudinal outcomes for each individual. Therefore, we did not include weights in this study of the time-varying SMART design. However, for increased complexity of time-varying SMART designs, weights may be incorporated into the analysis in a future study to develop more robust estimations and results.

## Conclusion

The proposed time-varying two-stage SMART design can take into account the time associated with the first-stage interventions and thus could result in clinical trials with fewer side effects and lower cost. Additionally, the two modeling approaches we proposed are able to provide good estimations of the means of the final outcomes of all the embedded interventions. The joint modeling approach resulted in more accurate estimates and higher estimated coverage probabilities; therefore, we recommend using joint modeling to analyze data generated from the time-varying designs proposed in this manuscript.

## Additional files

Additional file 1: Embedded adaptive interventions in the SMART design of Figs. 1 and 2. **Table S1**. Eight embedded adaptive interventions in the SMART design of Fig. 1. **Table S2.** Four embedded adaptive interventions in the SMART design of Fig. 2, word document. (DOCX 14 kb) Additional file 2: Conditional expectation of TVMEM, word document. (DOCX 44 kb) Additional file 3: Data organization and Implementation. **Table S3.** Longitudinal data organization, word document. (DOCX 61 kb) Additional file 4:
**Table S4.** The effect sizes associated with U_1_ and U_2_ influencing T_1_ and Y_1_ but not Y_2_. **Table S5.** The effect sizes associated with U_1_ and U_2_ influencing T_1_, Y_1_ and Y_2_. **Table S6.** The effect sizes associated with U_1_ and U_2_ influencing T_1_ and Y_2_ but not Y_1_, word document. (DOCX 49 kb)
