# Supplementary material for: Time-varying SMART design and data analysis methods for evaluating adaptive intervention effects
Source: BMC Med Res Methodol. 2016 Aug 30;16(1):112. doi: 10.1186/s12874-016-0202-7 (PMC5006275; doi:10.1186/s12874-016-0202-7)
Supplement: Additional file 1: — Embedded adaptive interventions in the SMART design of Figs. 1 and 2. Table S1. Eight embedded adaptive interventions in the SMART design of Fig. 1. Table S2. Four embedded adaptive interventions in the SMART design of Fig. 2, word document. (DOCX 14 kb) [file 12874_2016_202_MOESM1_ESM.docx]

**Additional file 1: Embedded adaptive interventions in the SMART design of Figures 1 and 2**

**Table S1. Eight embedded adaptive interventions in the SMART design of Figure 1.**

|  | **(*A_1_,A_2R_,A_2NR_*)** | **Adaptive interventions** |
| --- | --- | --- |
| (B,B,B+) | (1,1,1) | First, offer behavioral intervention; then continue behavioral intervention for responders and increase the intensity of behavioral intervention for non-responders. |
| (B,B,B+M) | (1,1,-1) | First, offer behavioral intervention; then continue behavioral intervention for responders and add medication for non-responders. |
| (B,B-,B+) | (1,-1,1) | First, offer behavioral intervention; then decrease the intensity of behavioral intervention for responders and increase the intensity of behavioral intervention for non-responders. |
| (B,B-,B+M) | (1,-1,-1) | First, offer behavioral intervention; then decrease the intensity of behavioral intervention for responders and add medication for non-responders. |
| (M,M,M+) | (-1,1,1) | First, offer medication; then continue medication for responders and increase the intensity of medication for non-responders. |
| (M,M,B+M) | (-1,1,-1) | First, offer medication; then continue medication for responders and add behavioral intervention for non-responders. |
| (M,M-,M+) | (-1,-1,1) | First, offer medication; then decrease the intensity of medication for responders and increase the intensity of medication for non-responders. |
| (M,M-,B+M) | (-1,-1,-1) | First, offer medication; then decrease the intensity of medication for responders and add behavioral intervention for non-responders. |

*M: medication; B: behavioral intervention; M+: intensified medication; B+: intensified behavioral intervention; B+M: combined treatment of behavioral intervention and medication*

**Table S2. Four embedded adaptive interventions in the SMART design of Figure 2.**

|  | **(*A_1_, A_2NR_*)** | **Adaptive interventions** |
| --- | --- | --- |
| (B,B+) | (1, 1) | First, offer behavioral intervention; then continue behavioral intervention for responders and increase the intensity of behavioral intervention for non-responders. |
| (B,B+M) | (1, 1) | First, offer behavioral intervention; then continue behavioral intervention for responders and add medication for non-responders. |
| (M,M+) | (-1,1) | First, offer medication; then continue medication for responders and increase the intensity of medication for non-responders. |
| (M,B+M) | (-1,-1) | First, offer medication; then continue medication for responders and add behavioral intervention for non-responders. |
