# Supplementary material for: Time-varying SMART design and data analysis methods for evaluating adaptive intervention effects
Source: BMC Med Res Methodol. 2016 Aug 30;16(1):112. doi: 10.1186/s12874-016-0202-7 (PMC5006275; doi:10.1186/s12874-016-0202-7)
Supplement: Additional file 2: — Conditional expectation of TVMEM, word document. (DOCX 44 kb) [file 12874_2016_202_MOESM2_ESM.docx]

**Additional file 2: Conditional expectation of TVMEM:**

**Models (1) - (4) can also be written in the form of conditional expectation as**

$$E[Y_{i}|A_{1i}(t),A_{2i}(t),t]=Z_{i}\eta(t)+\beta_{0}(t)+\beta_{1}(t)A_{1i}(t)+\beta_{2}(t)A_{2i}(t)+\beta_{3}(t)t+\beta_{4}(t)A_{1i}(t)\cdot A_{2i}(t)\begin{matrix} & (1) \end{matrix},$$

$$E[Y_{i}|t=0]=Z_{i}\eta_{0}+\beta_{00}\begin{matrix} \begin{matrix} \begin{matrix} & \end{matrix} & & & \end{matrix} & (2) \end{matrix},$$

$$E[Y_{i}|A_{1i},t=T_{1i}]=Z_{i}\eta_{1}+\beta_{01}+\beta_{11}A_{1i}+\beta_{31}T_{1i}(A_{1i})\begin{matrix} \begin{matrix} & \end{matrix} & (3) \end{matrix},$$

and

$$\begin{matrix} E[Y_{i}|A_{1i},A_{2Ri},A_{2NRi},t=T_{2i}]=Z_{i}\eta_{2}+\beta_{02}+\beta_{12}A_{1i}+\beta_{22}A_{2Ri}+\beta_{23}A_{2NRi}+ \\ \begin{matrix} \begin{matrix} \begin{matrix} \begin{matrix} & \end{matrix} & & \end{matrix} & & \end{matrix} & & & \end{matrix}\beta_{32}T_{2i}(A_{1i},A_{2Ri},A_{2NRi})+\beta_{41}A_{1i}\cdot A_{2Ri}+\beta_{42}A_{1i}\cdot A_{2NRi}\begin{matrix} & (4) \end{matrix}. \end{matrix}$$

**Accordingly, the conditional expectation of the final outcomes for each of the 8 embedded adaptive interventions in the SMART design of Figure 1 is**

$E[Y_{i}|1,1,1,t=T_{2i}]=Z_{i}\eta_{2}+\beta_{02}+\beta_{12}+\beta_{22}+\beta_{23}+\beta_{32}T_{2i}(1,1,1)+\beta_{41}+\beta_{42}$ for subjects following adaptive intervention (A_1_=1,A_2R_= 1,A_2NR_=1);

$E[Y_{i}|1,1,-1,t=T_{2i}]=Z_{i}\eta_{2}+\beta_{02}+\beta_{12}+\beta_{22}-\beta_{23}+\beta_{32}T_{2i}(1,1,-1)+\beta_{41}-\beta_{42}$ for subjects following adaptive intervention (A_1_=1,A_2R_= 1,A_2NR_=-1);

$E[Y_{i}|1,-1,1,t=T_{2i}]=Z_{i}\eta_{2}+\beta_{02}+\beta_{12}-\beta_{22}+\beta_{23}+\beta_{32}T_{2i}(1,-1,1)-\beta_{41}+\beta_{42}$ for subjects following adaptive intervention (A_1_=1,A_2R_= -1,A_2NR_=1);

$E[Y_{i}|1,-1,-1,t=T_{2i}]=Z_{i}\eta_{2}+\beta_{02}+\beta_{12}-\beta_{22}-\beta_{23}+\beta_{32}T_{2i}(1,-1,-1)-\beta_{41}-\beta_{42}$ for subjects following adaptive intervention (A_1_=1,A_2R_=- 1,A_2NR_=-1);

$E[Y_{i}|-1,1,1,t=T_{2i}]=Z_{i}\eta_{2}+\beta_{02}-\beta_{12}+\beta_{22}+\beta_{23}+\beta_{32}T_{2i}(-1,1,1)-\beta_{41}-\beta_{42}$ for subjects following adaptive intervention (A_1_=-1,A_2R_= 1,A_2NR_=1);

$E[Y_{i}|-1,1,-1,t=T_{2i}]=Z_{i}\eta_{2}+\beta_{02}-\beta_{12}+\beta_{22}-\beta_{23}+\beta_{32}T_{2i}(-1,1,-1)-\beta_{41}+\beta_{42}$ for subjects following adaptive intervention (A_1_=-1,A_2R_= 1,A_2NR_=-1);

$E[Y_{i}|-1,-1,1,t=T_{2i}]=Z_{i}\eta_{2}+\beta_{02}-\beta_{12}-\beta_{22}+\beta_{23}+\beta_{32}T_{2i}(-1,-1,1)+\beta_{41}-\beta_{42}$ for subjects following adaptive intervention (A_1_=-1,A_2R_= -1,A_2NR_=1);

$E[Y_{i}|-1,-1,-1,t=T_{2i}]=Z_{i}\eta_{2}+\beta_{02}-\beta_{12}-\beta_{22}-\beta_{23}+\beta_{32}T_{2i}(-1,-1,-1)+\beta_{41}+\beta_{42}$ for subjects following adaptive intervention (A_1_=-1,A_2R_= -1,A_2NR_=-1).

**The conditional expectation of the final outcomes for each of the 4 embedded adaptive interventions in the SMART design of Figure 2 is**

$E[Y_{i}|1,1,t=T_{2i}]=Z_{i}\eta_{2}+\beta_{02}+\beta_{12}+\beta_{23}+\beta_{32}T_{2i}(1,1)+\beta_{42}$ for subjects following adaptive intervention (A_1_=1 ,A_2NR_=1);

$E[Y_{i}|1,-1,t=T_{2i}]=Z_{i}\eta_{2}+\beta_{02}+\beta_{12}-\beta_{23}+\beta_{32}T_{2i}(1,-1)-\beta_{42}$ for subjects following adaptive intervention (A_1_=1, A_2NR_=-1);

$E[Y_{i}|-1,1,t=T_{2i}]=Z_{i}\eta_{2}+\beta_{02}-\beta_{12}+\beta_{23}+\beta_{32}T_{2i}(-1,1)-\beta_{42}$ for subjects following adaptive intervention (A_1_=-1,A_2NR_=1);

$E[Y_{i}|-1,-1,t=T_{2i}]=Z_{i}\eta_{2}+\beta_{02}-\beta_{12}-\beta_{23}+\beta_{32}T_{2i}(-1,-1)+\beta_{42}$ for subjects following adaptive intervention (A_1_=-1,A_2NR_=-1).
