# Supplementary material for: Time-varying SMART design and data analysis methods for evaluating adaptive intervention effects
Source: BMC Med Res Methodol. 2016 Aug 30;16(1):112. doi: 10.1186/s12874-016-0202-7 (PMC5006275; doi:10.1186/s12874-016-0202-7)
Supplement: Additional file 3: — Data organization and Implementation. Table S3. Longitudinal data organization, word document. (DOCX 61 kb) [file 12874_2016_202_MOESM3_ESM.docx]

**Additional file 3: Data organization and Implementation**

**Data organization**

As discussed in the Methods, both analytic approaches required a linear mixed model fitted in R by function “*lme( )”* in package *“nlme”*^29^. Data obtained from the design had to be organized longitudinally to apply this function. Therefore, we stacked the observed data at three time points {$0,T_{1},T_{2}$} and formed a new data set, as shown in Table A1. For each variable, the three parts of data in that column represent the corresponding measurements for all the subjects at time points {$0,T_{1},T_{2}$}, respectively. The first column is the subjects’ “**id**”, which is numbered from 1 to n (number of subjects), and thus the vector (1,2,…,n) is repeated for the three parts in that column. The variable “***Y***” in the second column is the longitudinal outcome of interest. Columns 3 to 14 represent the design matrix in models (2)–(4), and the corresponding coefficients are $(\beta_{0}^{Y_{0}},\beta_{0}^{Y_{1}},\beta_{0}^{Y_{2}},\eta,\beta_{1},\beta_{21},\beta_{22},\beta_{23},\beta_{31},\beta_{32},\beta_{41},\beta_{42})$. Data analyses were conducted with the data organized in Table A1 using the two proposed modeling approaches.

**Table S3.** Longitudinal data organization


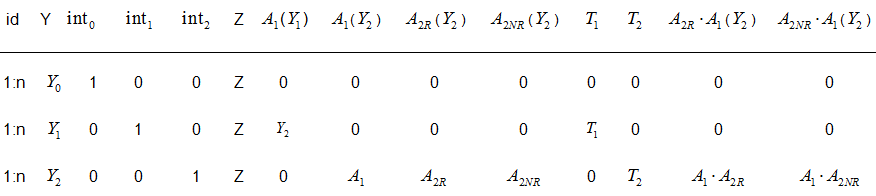


**Implementation**

Data analysis using the joint model was implemented in R with package “JM”^21^. A linear mixed model was fitted for the longitudinal outcomes $Y=(0,Y_{1},Y_{2})$ using function “*lme( )” ,* and a Cox model was fitted for the time to the event of interest, $T_{1}$. Then the outputs from these two models were supplied as the main arguments in function “*jointModel()*” for fitting the joint model, using the data in Table A1. (More specifically, the joint model fitted using “jointModel()” had exactly the same structure for the linear mixed effects and survival submodels as these two separately fitted models, with the addition that in the survival model the effect of the estimated “true” baseline longitudinal outcome $\mathrm{m}_{i}(0)$ was included in the linear predictor.) This function approximates the integral $\int p(\mathrm{T}1{}_{i},\delta_{i}|b_{i};\beta,\gamma,\alpha,\eta)[\prod_{j} p\{\mathrm{Y}_{i}(\mathrm{t}_{ij})|\mathrm{b}_{i};\beta,\eta\}]\mathrm{p}(\mathrm{b}_{i};\sigma_{b})\mathrm{db}$using either the Gauss-Hermite rule or the fully exponential Laplace approximation and the integral $\int_{0}^{T1_{i}} \mathrm{h}_{i}(s|\mathrm{m}_{i}(0);\beta,\gamma,\alpha,\eta)\mathrm{ds}$ in the likelihood model of $T1_{i}$ using the Gauss-Kronrod rule. The maximization of the log-likelihood is based on a hybrid optimization procedure, which starts with the expectation-maximization algorithm for a fixed number of iterations, and if convergence is not achieved, it switches to a quasi-Newton algorithm (method “BFGS”^30-32^ in R function “optim()”^33^) until convergence is attained.

For all these options, the linear predictors in model (5) were written as $\eta_{i}=\gamma_{0}+\gamma_{1}A1_{i}+$ $\gamma_{2}W_{i}+$ $\alpha\mathrm{m}_{i}\{\max(\mathrm{t}-\mathrm{k},0)\}$ when we let *“parameterization = "value"”* in the function of *“jointModel()”.* Because in our example, only the underlying baseline measurement $\mathrm{m}_{i}(0)$was included in model (5), the linear predictor above should satisfy $\mathrm{m}_{i}\{\max(\mathrm{t}-\mathrm{k},0)\}=\mathrm{m}_{i}(0)$, which indicates that $\max(\mathrm{t}-\mathrm{k},0)=0$ and $(\mathrm{t}-\mathrm{k})\leq0$. In this expression, *“t”* is specified by the *“timeVar”* argument, which refers to the time variable and *“k”* is specified by the *“lag”* argument, denoting a lag effect in the time-dependent covariate represented by the TVMEM in the function *“jointmodel()”.* Therefore, we set “timeVar” equal to “$T_{1}$” and “lag” equal to any value larger than $t_{10}$ (e.g., in the simulations, we set “lag=2” as $t_{10}$=1). In this way, we had max(t-k,0)=0 for all the subjects and thus only the baseline longitudinal measurement was included in the survival submodel. Moreover, we set **method = "weibull-PH-GH"** in the function to apply the Weibull relative risk model in the regression of $T_{1}$.
