# Supplementary material for: Time-varying SMART design and data analysis methods for evaluating adaptive intervention effects
Source: BMC Med Res Methodol. 2016 Aug 30;16(1):112. doi: 10.1186/s12874-016-0202-7 (PMC5006275; doi:10.1186/s12874-016-0202-7)
Supplement: Additional file 4: — Table S4. The effect sizes associated with U1 and U2 influencing T1 and Y1 but not Y2. Table S5. The effect sizes associated with U1 and U2 influencing T1, Y1 and Y2. Table S6. The effect sizes associated with U1 and U2 influencing T1 and Y2 but not Y1, word document. (DOCX 49 kb) [file 12874_2016_202_MOESM4_ESM.docx]

**Additional file 4: Influence of unmeasured confounders**

We simulated two unmeasured variables, U_1_ and U_2_ that affect the outcomes but were not included in the analyses.

**Table S4. The effect sizes associated with U_1_ and U_2_ influencing T_1_ and Y_1_ but not Y_2_**

**Panel A: The coefficients of unobserved confounders in the simulation model**

| \|  \| **U_1_** \| **U_2_** \| \| --- \| --- \| --- \| \| **T_1_** \| 0.2 \| -0.3 \| \| **Y_1_** \| -0.4 \| 0.3 \| \| **Y_2_** \| 0 \| 0 \| \|  \|  \|  \| | | | | | | |  |  |
| --- | --- | --- | --- | --- | --- | --- | --- | --- |
| **Panel B: Parameter estimation** | | | | | | |  |  |
|  | | | | $\beta_{12}$ | $\beta_{22}$ | $\beta_{23}$ | | $\beta_{32}$ |
|  | | | | (first-stage interventions$A_{1}$) | (second-stage interventions for responders$A_{2R}$) | (second-stage interventions for non-responders$A_{2NR}$) | | (time of intervention $T_{2}$) |
|  | | | |  |  |  | |  |
| **True value** | | | | **- 0.4** | **0.5** | **0.4** | | **2.0** |
| Joint  Model | | | Estimate | -0.391 | 0.515 | 0.389 | | 1.909 |
|  |  |  | MSE | 0.011 | 0.033 | 0.016 | | 0.134 |
|  |  |  | CI% | 98% | 94% | 96% | | 97% |
|  |  |  | Length of CI | 0.476 | 0.688 | 0.530 | | 1.464 |
|  |  | |  |  |  | |  |  |
|  | TVMEM | Estimate |  | -0.508 | 0.513 | 0.389 |  | 4.010 |
|  |  | MSE |  | 0.023 | 0.033 | 0.017 |  | 4.165 |
|  |  | CI% |  | 89.5% | 94% | 96% |  | 0% |
|  |  | Length of CI |  | 0.482 | 0.706 | 0.541 |  | 1.446 |

**Table S5. The effect sizes associated with U_1_ and U_2_ influencing T_1_, Y_1_ and Y_2_**

**Panel A: coefficients of unobserved confounders in the simulation model**

| \|  \| **U_1_** \| **U_2_** \| \| --- \| --- \| --- \| \| **T_1_** \| 0.2 \| -0.3 \| \| **Y_1_** \| -0.4 \| 0.3 \| \| **Y_2_** \| -0.2 \| -0.3 \| | | | | | | |  |  |
| --- | --- | --- | --- | --- | --- | --- | --- | --- |
| **Panel B: coefficients of unobserved confounders** | | | | | | |  |  |
|  | | | | $\beta_{12}$ | $\beta_{22}$ | $\beta_{23}$ | | $\beta_{32}$ |
|  | | | | (first-stage interventions$A_{1}$) | (second-stage interventions for responders$A_{2R}$) | (second-stage interventions for non-responders$A_{2NR}$) | | (time of intervention $T_{2}$) |
|  | | | |  |  |  | |  |
| **True value** | | | | **- 0.4** | **0.5** | **0.4** | | **2.0** |
| Joint  Model | | | Estimate | -0.391 | 0.513 | 0.396 | | 1.638 |
|  |  |  | MSE | 0.010 | 0.037 | 0.015 | | 0.218 |
|  |  |  | CI% | 98% | 96% | 97% | | 92% |
|  |  |  | Length of CI | 0.476 | 0.689 | 0.529 | | 1.458 |
|  |  | |  |  |  | |  |  |
|  | TVMEM | Estimate |  | -0.505 | 0.515 | 0.395 |  | 3.731 |
|  |  | MSE |  | 0.021 | 0.037 | 0.015 |  | 3.090 |
|  |  | CI% |  | 90% | 95% | 97% |  | 1% |
|  |  | Length of CI |  | 0.482 | 0.707 | 0.539 |  | 1.441 |

**Table S6. The effect sizes associated with U_1_ and U_2_ influencing T_1_ and Y_2_ but Y_1_**

**Panel A: coefficients of unobserved confounders in the simulation model**

|  | **U_1_** | **U_2_** |
| --- | --- | --- |
| **T_1_** | 0.3 | -0.4 |
| **Y_1_** | 0 | 0 |
| **Y_2_** | 0.2 | -0.3 |

| **Panel B: coefficients of unobserved confounders** | | | | | | |  |  |
| --- | --- | --- | --- | --- | --- | --- | --- | --- |
|  | | | | $\beta_{12}$ | $\beta_{22}$ | $\beta_{23}$ | | $\beta_{32}$ |
|  | | | | (first-stage interventions$A_{1}$) | (second-stage interventions for responders$A_{2R}$) | (second-stage interventions for non-responders$A_{2NR}$) | | (time of intervention $T_{2}$) |
|  | | | |  |  |  | |  |
| **True value** | | | | **- 0.4** | **0.5** | **0.4** | | **2.0** |
| Joint  Model | | | Estimate | -0.420 | 0.490 | 0.408 | | 2.210 |
|  |  |  | MSE | 0.012 | 0.035 | 0.015 | | 0.163 |
|  |  |  | CI% | 99% | 91% | 96% | | 94% |
|  |  |  | Length of CI | 0.477 | 0.696 | 0.529 | | 1.463 |
|  |  | |  |  |  | |  |  |
|  | TVMEM | Estimate |  | -0.531 | 0.491 | 0.410 |  | 4.263 |
|  |  | MSE |  | 0.028 | 0.037 | 0.016 |  | 5.232 |
|  |  | CI% |  | 83% | 91% | 96% |  | 0% |
|  |  | Length of CI |  | 0.483 | 0.714 | 0.539 |  | 1.439 |
